# Effect of internet-delivered exposure therapy versus healthy lifestyle promotion for patients with persistent physical symptoms (SOMEX1): a randomized controlled trial with planned moderator analysis

**DOI:** 10.1017/S0033291725101244

**Published:** 2025-08-08

**Authors:** Jonna Hybelius, Sandra af Winklerfelt Hammarberg, Sigrid Salomonsson, Caroline Wachtler, Majken Epstein, Anna Olsson, Emma Strand, Lina Söderström Winter, Tomas Åkerlund, Daniel Björkander, Amanda Kosic, Gabriel Chahin, John Wallert, Eva Toth-Pal, Steven Nordin, Erland Axelsson

**Affiliations:** 1 Division of Family Medicine and Primary Care, Department of Neurobiology, Care Sciences and Society, Karolinska Institutet, Stockholm, Sweden; 2 Liljeholmen University Primary Health Care Centre, Academic Primary Health Care Centre, Region Stockholm, Stockholm, Sweden; 3 Centre for Psychiatry Research, Department of Clinical Neuroscience, Karolinska Institutet & Stockholm Health Care Services, Region Stockholm, Stockholm, Sweden; 4 Division of Psychology, Department of Clinical Neuroscience, Karolinska Institutet, Stockholm, Sweden; 5 School of Law, Psychology and Social Work, Örebro University, Örebro, Sweden; 6 Department of Psychology, Umeå University, Umeå, Sweden

**Keywords:** exposure therapy, healthy lifestyle promotion, internet-based, moderator analysis, persistent physical symptoms, primary care, randomized controlled trial, transdiagnostic

## Abstract

**Background:**

The management of persistent physical symptoms poses a challenge in many healthcare settings, including primary care. Psychological treatments that involve exposure have shown promise for several conditions where patients suffer from persistent physical symptoms and unwanted responses to these. It is unclear, however, to what extent exposure therapy has effects beyond existing routine care interventions and who benefits the most.

**Methods:**

A randomized controlled trial at a primary care center in Stockholm, Sweden compared 10 weeks of internet-delivered exposure therapy (*n* = 80) to healthy lifestyle promotion (HLP; *n* = 81) for patients bothered by at least one persistent physical symptom. The primary outcome was the mean reduction in subjective somatic symptom burden (Patient Health Questionnaire 15) as measured week-by-week up to the post-treatment assessment. Secondary outcomes included symptom preoccupation, anxiety, depression symptoms, and functional impairment.

**Results:**

Patients contributed 1544 datapoints during treatment. The primary analysis showed no significant advantage of exposure therapy versus HLP in the reduction of mean somatic symptom burden (*d* = 0.14; *p* = 0.220). In secondary analyses, exposure showed superiority in the reduction of symptom preoccupation (*d* = 0.31; *p* = 0.033) but not anxiety, depression symptoms, or functional impairment. A higher somatic symptom burden or symptom preoccupation before treatment was predictive of a larger advantage of exposure versus HLP.

**Conclusions:**

Exposure therapy does not appear to show noteworthy average benefit over HLP, with the exception of symptom preoccupation. Substantial benefits are seen in patients with very high symptom burden or symptom preoccupation.

## Introduction

*Persistent physical symptoms* is an umbrella term for somatic complaints that persist over time and give rise to distress, regardless of etiology (Löwe et al., 2024). Common complaints such as gastrointestinal symptoms, fatigue, and pain account for up to half of primary care consultations, can be difficult to classify, and often defy straightforward medical explanation (Burton et al., 2020; Haller, Cramer, Lauche, & Dobos, 2015; Henningsen, Zipfel, Sattel, & Creed, 2018). Relatively well-defined conditions, such as cancer disease, cardiovascular disease, and inflammatory bowel disease, are also associated with persistent symptoms and associated distress, even after standard medical treatment (Emery et al., 2022; Halpin & Ford, 2012; Henningsen et al., 2018; Kohlmann et al., 2013). Merely the subset of conditions where persistent physical symptoms lack a clear medical explanation are associated with societal costs comparable to major depression or anxiety disorders (Konnopka et al., 2012).

In routine clinical care, patients with persistent physical symptoms are offered various clinical interventions, but these often have modest effects (Henningsen et al., 2018; Kleinstauber et al., 2014; Swainston et al., 2023). One common example is the promotion of healthy lifestyle behaviors, such as physical activity, sleep hygiene, and a healthy diet (Toussaint et al., 2025). Lack of physical activity is a robust predictor of somatic disease (Lee et al., 2012) and adverse mental health outcomes (Kim et al., 2012; Noetel et al., 2024). Similarly, disturbed sleep is associated with symptom severity and poor quality of life in patients with persistent physical symptoms (Ionescu et al., 2021). Broad interventions to promote a healthy lifestyle can have beneficial effects (Zhang et al., 2024), but patients with persistent physical symptoms also describe unmet needs (Houwen et al., 2017; Taylor et al., 2016).

Exposure therapy is a treatment where patients voluntarily engage in activities that give rise to symptoms or associated distress to achieve therapeutic effects. Psychological treatments encompassing exposure have shown promise both when delivered face-to-face and in a guided online format for various conditions characterized by persistent physical symptoms, such as chronic pain, functional somatic syndromes, and common somatic diseases like asthma and atrial fibrillation (Axelsson et al., 2023; Boersma et al., 2019; Hedman-Lagerlöf et al., 2024; Parry et al., 2012; Särnholm et al., 2023; Woods & Asmundson, 2008). Even so, in primary care, therapies based on exposure are rarely administered, perhaps because existing protocols are tailored for specific symptom domains (e.g., gastrointestinal symptoms or pain only). In light of evidence suggesting that various forms of symptom preoccupation (i.e., the tendency to respond strongly to and engage in behaviors contingent on somatic symptoms), including hypervigilance and avoidance behaviors, are common across subtypes of persistent physical symptoms (Löwe et al., 2022), a transdiagnostic treatment approach could be a viable option. Addressing several domains in the same treatment could potentially both aid dissemination and benefit patients, considering the sizeable overlap between conditions characterized by elevated somatic symptom burden (Fink et al., 2007).

We recently piloted a transdiagnostic exposure therapy that can be tailored to patients with a wide range of persistent physical symptoms and unwanted responses to those symptoms. In the feasibility trial (*N* = 33), patients rated the treatment as credible, were adherent, and reported large and sustained reductions in somatic symptom burden (*d* = 0.90) and symptom preoccupation (*d* = 1.17) (Hybelius et al., 2022). It is unclear, however, whether exposure therapy offers added benefits compared to more common interventions, such as healthy lifestyle promotion. It is also unclear which patients benefit the most from exposure. Therefore, we conducted a randomized controlled trial in primary care that compared internet-delivered exposure therapy with healthy lifestyle promotion for patients with persistent physical symptoms. We hypothesized that exposure therapy would have superior effects on somatic symptom burden (primary outcome) and symptom preoccupation, general anxiety, depression, and functional impairment (secondary outcomes). Furthermore, based on theoretical models of persistent physical symptoms (e.g., Meulders, 2020; Olatunji et al., 2011; Rief & Barsky, 2005; Wolters, Peerdeman, & Evers, 2019) that emphasize the role of conditioned responses and the detrimental effects of symptom preoccupation – such as avoidance, which is typically targeted in exposure-based treatment – we hypothesized that higher levels of pretreatment somatic symptom burden and symptom preoccupation would predict a larger advantage of exposure therapy.

## Method

### Design

This was a randomized controlled trial of internet-delivered exposure therapy (*n* = 80) versus healthy lifestyle promotion (*n* = 81) for adult patients with persistent physical symptoms. The trial was based at Liljeholmen University Primary Health Care Centre in collaboration with Karolinska Institutet, Stockholm, Sweden. Simulations informed by the feasibility study (Hybelius et al., 2022) indicated that power in the primary analysis was 80% to test for an effect in the lower moderate range (*d* = 0.40) with up to 20% data loss. The trial was approved by the Swedish Ethical Review Authority on May 26, 2021 (2021-01400), preregistered at ClinicalTrials.gov on June 18, 2021 (NCT04942028), and is reported in accordance with the CONSORT guidelines (Supplementary File 02).

### Recruitment

General practitioners at the primary care center were encouraged to identify patients with elevated somatic symptom burden and potential interest in a behavioral intervention. Around May 2022, inflow to the trial was found unsatisfactory, and per the preregistered protocol, a hybrid strategy was adopted where we also advertised more broadly, primarily via social media. These advertisements focused on applicants being bothered by persistent physical symptoms and framed participation as a means of potentially achieving beneficial health outcomes. Applicants provided informed consent and completed screening questionnaires via an encrypted web measurement platform (‘*BASS*’). A structured eligibility interview was held with a mental health clinician aided by the Mini-International Neuropsychiatric Interview (Sheehan et al., 1998) and Health Preoccupation Diagnostic Interview (Axelsson et al., 2016).

The eligibility criteria (details in Supplementary Table DS1 and Supplementary Methods) required participants to be either ‘much bothered’ by at least one physical symptom (item of the Patient Health Questionnaire 15 [PHQ-15]) or report a moderate somatic symptom burden (PHQ-15 ≥ 10), with a duration of at least 4 months. Physical symptoms were not required to be medically unexplained, could belong to any domain, and could be associated with any combination of unwanted emotions. Comorbidities were allowed, but similar to the bodily distress disorder diagnosis of the International Classification of Diseases 11 (World Health Organization, 2024), the dominant clinical problem was required not to be best explained as primary pathological health anxiety (pha) or a non-somatoform psychiatric disorder. Participants had to live in Stockholm County because this was the catchment area of the primary care center. Participants also had to express interest in psychological treatment, be 18 years or older, be fluent in Swedish, and not meet criteria for a serious psychiatric disorder or report severe suicidal ideation. In collaboration with a general practitioner, applicants’ medical status was assessed to rule out medical obstacles to exposure therapy. Such could include a need for further investigation of alarm symptoms, ongoing diagnosis-specific treatment, or physical activity being potentially harmful. Continuous psychotropic medication had to be either non-existent or stable for ≥4 weeks, and, in the case of alcohol or substance use, this could not risk severely interfering with treatment. Participants could not have planned an absence more than 1 week during treatment and were enrolled when the pre-treatment assessment had been completed. Recruitment ended once the target sample size of 160 was reached and all scheduled eligibility interviews had been completed.

### Randomization

Patients were randomized (1:1) to 10 weeks of exposure therapy or healthy lifestyle promotion. This was done in consecutive cohorts, using a true random number service (www.random.org), by an independent assistant without access to patient characteristics. The decision to include a patient was taken prior to randomization. Clinicians could not foresee future allocations or match patients to treatments.

### General treatment framework

Both interventions were delivered in a guided online format via the public healthcare web platform of Stockholm (‘*1177 Stöd-och behandling*’*)* and were accessible from any web-enabled device. The content was divided into 10 text-based modules, with simple illustrations. Ending each module, the patient answered questions for reflection, focusing on the content and treatment progress. Access to new modules was given contingent on progress, with a recommended pace of one module per week. Communication with a therapist transpired via email-like asynchronous messages. After completion of each module, or at least once per week, the therapist provided written feedback on the patient’s work. Patients could freely contact their therapist and expect a written reply within two working days. If a patient had been inactive for more than a few days, therapists attempted to make contact via telephone, aiming to steer the patient back to the web platform. No face-to-face or video sessions were included in any of the treatments, the rationale for this being that guided internet-delivered therapy typically has produced effects comparable to those of treatment delivered face-to-face in randomized controlled trials (Axelsson et al., 2020; Hedman-Lagerlöf et al., 2023). The nine therapists who administered treatment were psychologists or psychotherapists, with half a year to 8 years of prior experience, working at the study site (Supplementary Table DS2). All received weekly supervision by the first or last author. All patients had access to standard routine care.

### Exposure therapy

Unlike many other therapies that incorporate exposure as one of several components, this protocol (Open Science Framework identifier: cnbwj) focused solely on exposure and response prevention and the elements necessary for working with these strategies, such as a diary for identifying relevant situations and behaviors. The rationale for exposure was that heightened reactivity to physical symptoms – including unwanted emotions, changes in attention, and physiological arousal – is likely to lead to a higher subjective somatic symptom burden, and that exposure is a method for changing such responses, reducing the need for compensatory strategies, and promoting long-term wellbeing. The patient was encouraged to approach situations that give rise to distress (exposure) while refraining from strategies aimed at short-term relief (response prevention), on a daily basis. For example, a patient with gastrointestinal complaints who avoided eating out for fear of stomach pains could practice doing so, while refraining from checking the menu in advance and devoting excessive attention to exits and physical sensations. Three fictitious exemplar patients were presented: one suffering from pain and fatigue, one with a heart condition, and one with anxiety about gastrointestinal symptoms. Therapists were encouraged to support each participant in tailoring exposure exercises for their specific needs. This entailed approaching a broad spectrum of situations that gave rise to either symptoms or symptom-related distress and unwanted emotional responses such as fear, shame, or anger. See Supplementary Table DS3 for an overview of the protocol.

### Healthy lifestyle promotion

Although no criterion standard treatment for all patients with persistent physical symptoms exists, healthy lifestyle promotion is commonly offered and typically includes support from a clinician, a rationale for behavioral change, and systematic evaluation of symptoms and behaviors (Kettle et al., 2022; Toussaint et al., 2025; Zhang et al., 2024). The protocol was developed for this trial but aligned with existing information provided at the primary care center and via the national Swedish healthcare information platform. The text emphasized that improved lifestyle behaviors benefit overall physical and mental health, including persistent physical symptoms and related distress. Patients were encouraged to formulate clear and attainable goals and to keep a symptom diary on a daily basis (details in Supplementary Table DS3). Because we expected a higher average effect of exposure therapy, for ethical reasons, patients were offered to be crossed over to exposure therapy after the post-treatment assessment.

### Validated symptom outcomes

Patients completed self-report questionnaires via the web measurement platform before, weekly during, and after treatment. Those in exposure therapy also completed two follow-up assessments, 6 and 12 months after treatment. The primary outcome was somatic symptom burden measured using the PHQ-15 (Kroenke, Spitzer, & Williams, 2002) up to the post-treatment assessment. The PHQ-15 probes whether the respondent has been bothered by common somatic symptoms, such as back pain, stomach pain, or shortness of breath and exhibits adequate measurement properties (Hybelius et al., 2024). In this trial, to enable week-by-week measurements, the conventional 4-week version was administered at screening only, and a revised 1-week version was used at later time points. Secondary outcomes included symptom preoccupation measured weekly with a 1-week version of the Somatic Symptom Disorder–B Criteria Scale (SSD-12) (Toussaint et al., 2016) and pre- to post-treatment assessment of general anxiety with the GAD-7 (Spitzer, Kroenke, Williams, & Löwe, 2006), depression symptoms with the Patient Health Questionnaire-9 (PHQ-9) (Kroenke, Spitzer, & Williams, 2001), and functional impairment with the 12-item self-report World Health Organization Disability Assessment Schedule (WD2-12) (Üstün et al., 2010). In analyses that included both the PHQ-15 and the PHQ-9, only items 1 and 2 of the PHQ-9 (i.e., the core symptoms of depression: low mood and anhedonia) were used to reduce overlap between the scales.

### Process-related outcomes

Completion was defined as having initiated at least 5 out of 10 modules. In exposure, patients also reported the number of exposure exercises completed each week. Patients were considered dropouts if they (i) stopped replying for at least 3 weeks without resuming treatment or (ii) expressed the wish to discontinue treatment and did so. Treatment credibility was assessed using a five-item version of the Credibility/Expectancy scale (C/E scale) (Borkovec & Nau, 1972), and the strength of the working alliance using a six-item version of the Working Alliance Inventory (Hatcher & Gillaspy, 2006), both at week 3. After treatment, satisfaction was measured using the Client Satisfaction Questionnaire (CSQ-8) (Kelly et al., 2018), and patients were asked to report whether they had experienced adverse events (details in Supplementary Table DS12).

### Statistical analysis

Analyses were conducted in R 4.4.1 and Stata 15.1, in accordance with a preregistered statistical analysis plan (ClinicalTrials.gov identifier NCT04942028) that adhered to best practice guidelines (Gamble et al., 2017). The pre- to post-treatment (primary endpoint) effect outcomes were analyzed by an individual blind to treatment condition. To enable intention-to-treat analyses, missing data were multiply imputed using chained equations in mice 3.16.0 (van Buuren & Groothuis-Oudshoorn, 2011). Change in the symptom outcomes was analyzed using linear mixed effects regression. Models included the fixed effects of condition (exposure versus healthy lifestyle promotion), time, the condition × time interaction (main focus of tests), a random intercept and slope (time, within participants), and an autoregressive residual covariance structure (AR1). The time × time interaction was also added for the PHQ-15 and SSD-12, which improved model fit. Standardized mean effects (Cohen’s *d*) were obtained by dividing model-implied means by the full sample endpoint standard deviation.

Potential moderators of the controlled effect of exposure therapy on somatic symptom burden (PHQ-15) and symptom preoccupation (SSD-12) were tested based on three way time × group × moderator interactions in models that included a random intercept but not a random slope, for the following pre-treatment variables: overall somatic symptom burden, symptom preoccupation, depression symptoms, functional impairment, medically explained versus unexplained symptoms, recruitment path, age, gender, educational level, chronicity (i.e., years with physical symptoms), and treatment completer status. All potential moderators had inter-correlations <0.60. Response and deterioration were evaluated using preregistered definitions, and these dichotomous outcomes were analyzed using logistic regression (details in Supplementary Methods).

## Results

### Sample characteristics

**Figure 1.**
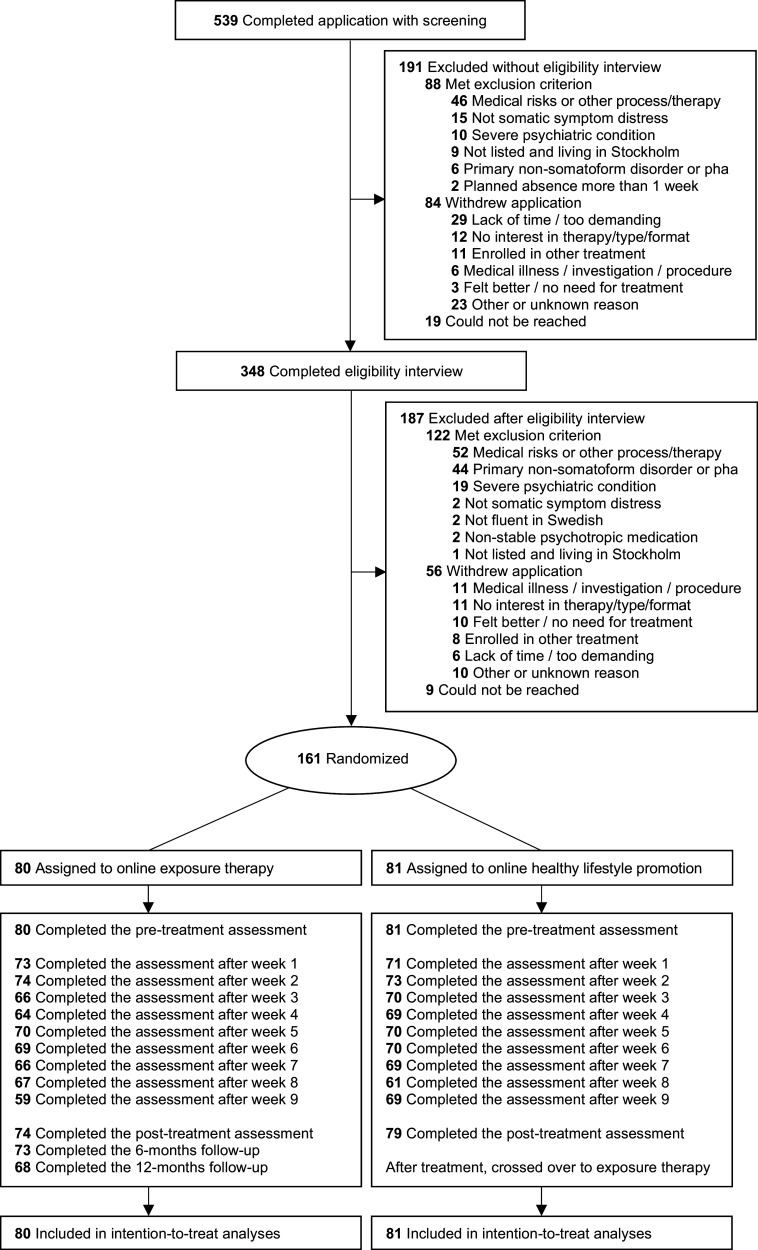
CONSORT flow diagram.

Recruitment lasted from October 7, 2021 (first enrollment) to October 7, 2023. The last 12-month follow-up was completed on January 2, 2025. The patient flow is presented in Figure 1. The average patient was a 48-year-old female recruited via social media with a tertiary education, moderate somatic symptom burden (screening PHQ-15: *M* = 13.0, *SD* = 4.8), and bothered by physical symptoms for 11.9 years (*SD* = 11.0). For additional characteristics, see Table 1.

**Table 1.** Patient characteristics before treatment

	Exposure therapy	Healthy lifestyle promotion
	(*n* = 80)	(*n* = 81)
*Sociodemographics*		
Age, mean (*SD*), median (range)	47.9 (12.4), 51.5 (22–73)	48.4 (11.9), 50 (20–72)
Female, *n* (%)	67 (84%)	70 (86%)
Postsecondary education, *n* (%)	62 (78%)	68 (84%)
Married or de facto, *n* (%)	50 (63%)	59 (73%)
Parent, *n* (%)	59 (74%)	54 (67%)
Occupation, *n* (%)		
Employed	61 (76%)	64 (79%)
Unemployed	7 (9%)	2 (2%)
Retired	3 (4%)	5 (6%)
Student	4 (5%)	4 (5%)
Disability pension	2 (3%)	3 (4%)
Other or unclear	3 (4%)	3 (4%)
*Clinical characteristics*		
PPS duration in years, mean (*SD*), median (range)	11.4 (9.2), 9.5 (0.5–46)	12.4 (12.5), 8 (1–57)
DSM–5 psychiatric disorders, *n* (%)		
Somatic symptom disorder	37 (46%)	32 (40%)
Major depressive disorder	16 (20%)	22 (27%)
At least one anxiety disorder^a^	21 (26%)	24 (30%)
Five most frequent non-DSM conditions (any time)^b^, *n* (%)		
Irritable bowel syndrome	33 (41%)	26 (32%)
Exhaustion disorder^c^	24 (30%)	30 (37%)
Osteoarthritis	24 (30%)	25 (31%)
Hypertension	22 (28%)	18 (22%)
Asthma	19 (24%)	16 (20%)
Symptom scales, mean (*SD*), median (range)		
Somatic symptom burden (PHQ–15)	13.1 (4.9), 12.5 (2–30)	12.9 (4.7), 13 (3–27)
Cardiopulmonary symptoms	0.6 (0.5), 0.5 (0–2)	0.5 (0.4), 0.5 (0–2)
Fatigue symptoms	1.3 (0.6), 1.5 (0–2)	1.4 (0.6), 1.5 (0–2)
Gastrointestinal symptoms	1.2 (0.6), 1.3 (0–2)	1.2 (0.7), 1.3 (0–2)
Pain symptoms	1.1 (0.5), 1.3 (0–2)	1.1 (0.6), 1 (0–2)
Symptom preoccupation (SSD–12)	26.7 (7.9), 25.5 (8–45)	25.9 (7.8), 25 (8–42)
General anxiety (GAD–7)	6.2 (3.9), 5.5 (0–20)	5.7 (4.7), 4 (0–19)
Depression core symptoms (PHQ–2)	1.4 (1.3), 1 (0–4)	1.8 (1.6), 2 (0–6)
Functional impairment (WD2–12)	24.2 (16.7), 22.9 (2.1–68.8)	26.7 (16.2), 25 (0.0–60.4)
Referred via routine care, *n* (%)	14 (18%)	28 (35%)
Psychotropic medication, *n* (%)	35 (44%)	38 (47%)
Experience of psychological treatment, *n* (%)	55 (70%)	59 (73%)

*Note*: PHQ-2, Patient Health Questionnaire 2 (scored 0–6); PHQ-15, Patient Health Questionnaire 15 (conventional 4-week version; scored 0–30, with domain subscales scored 0–2); PPS, persistent physical symptoms; SSD-12, Somatic Symptom Disorder B-criteria scale 12 (conventional non-specific timeframe version; scored 0–48); WD2-12, 12-item self-report World Health Organization Disability Assessment Schedule 2 (scored 0–100).

a Not including obsessive-compulsive disorder or post-traumatic stress disorder.

b Self-reported, pertaining to a diagnosis received at any point in time prior to this clinical trial.

c Exhaustion disorder is a diagnosis used in Swedish healthcare to characterize a chronic stress condition similar to clinical burnout, with marked exhaustion, impaired cognitive abilities, and a range of physical symptoms (Lindsäter et al., 2022). This was never the primary condition at enrollment.

### Missing data, adherence, and procedural outcomes including healthcare consumption

All patients completed the pre-treatment assessment. The post-treatment assessment was completed by 74 (74/80, 93%) in exposure therapy and 79 (79/81, 98%) in healthy lifestyle promotion. Two-thirds completed exposure therapy (53/80; 66%), and four-fifths completed healthy lifestyle promotion (65/81; 80%). The mean treatment credibility rating was higher in exposure therapy (*M* = 31.9, *SD* = 7.5 versus *M* = 24.0, *SD* = 9.8; *p* < 0.001). Therapists spent an average of 15.2 minutes per patient per week in exposure (*SD* = 8.7), versus 10.7 in healthy lifestyle promotion (*SD* = 5.4; *p* < 0.001). Healthcare consumption was comparable between the treatments. See Table 2 for details.

**Table 2.** Procedural outcomes pertaining to patient engagement, interaction with the therapist, and healthcare consumption alongside the trial

Variable	Exposure therapy	Healthy lifestyle promotion	Difference
	*M* (*SD*), median; range/*n* (%)	*M* (*SD*), median; range/*n* (%)	est (95% CI)
*Patient engagement with the treatment*			
Modules initiated out of 10	6.4 (3.0), 7; 1–10/*n* = 80	7.2 (2.9), 8; 1–10/*n* = 81	
Completed the treatment (initiated ≥5 modules)^a^	53/80 (66%)	65/81 (80%)	OR = 0.48 (0.23, 0.98)
Dropped out (≥3 weeks of inactivity, or explicit)	15/80 (19%)	12/81 (15%)	
Average weekly reported exposure exercises	5.8 (4.5), 4.9; 0.1–20.4/*n* = 75	Not applicable	
Credibility/expectancy (C/E scale) at week 3	31.9 (7.5), 34; 14–47/*n* = 71	24.0 (9.8), 23; 0–45/*n* = 76	Δ*M* = 8.0 (5.1, 11.0)
*Interaction with the therapist*			
Strength of working alliance (WAI–6) at week 3	33.0 (7.2), 33; 15–42/*n* = 71	27.3 (10.1), 28; 6–42/*n* = 76	Δ*M* = 5.9 (2.9, 8.8)
Therapist platform time, min per patient and week	15.2 (8.7), 13.8; 1.1–52.1/*n* = 78	10.7 (5.4), 9.9; 1.7–23.5/*n* = 79	Δ*M* = 4.3 (2.0, 6.5)
Therapist phone time, min per patient and week	2.0 (2.6), 1.5; 0–13.1/*n* = 80	1.2 (2.1), 0.4; 0–10.9/*n* = 81	
Messages sent by patient, total treatment	12.3 (10.9), 9.5; 0–55/*n* = 74	6.7 (4.5), 6; 0–27/*n* = 81	
Messages sent by therapist, total treatment	19.5 (7.8), 19; 6–40/*n* = 74	16.7 (5.9), 17; 3–35/*n* = 81	
*Healthcare consumption alongside the trial* ^b^			
Changes to psychotropic medication			
Initiated new medication	0/74 (0%)	1/79 (1%)	
Changed dosage in existing medication	4/74 (5%)	3/79 (4%)	
Terminated existing medication	0/74 (0%)	1/79 (1%)	
Met with general practitioner	7/74 (9%)	6/79 (8%)	
Met with psychologist or psychotherapist	7/74 (9%)	7/79 (9%)	

*Note*: Confidence intervals pertaining to differences between the two conditions were derived from the multiply imputed data. C/E scale, 5-item credibility/expectancy scale (theoretical range: 0–50); NNT, number needed to treat; WAI-6, 6-item working alliance inventory (theoretical range: 6–42).

a This result was identical when the analysis was repeated with the added requirement of ≥3 exposure exercises in the exposure treatment.

b These estimates concern healthcare consumption during the 10-week pre- to post-treatment main phase of the trial.

### Primary outcome: somatic symptom burden

In the primary analysis, exposure therapy was not superior to healthy lifestyle promotion in its average effect on somatic symptom burden (*b* = −0.7 [−1.8 to 0.4]; *p* = 0.220; *d* = 0.14; Table 3). Subscales and subgroup analyses are reported in Supplementary Tables DS4–DS5. In secondary analyses, the odds of achieving a minimal clinically important improvement (i.e., a decrease of at least 3 on the PHQ-15 [Hybelius et al., 2024]) was higher in exposure therapy (47/74 [64%] versus 35/79 [44%]; OR = 2.17 [1.36 to 3.46]). The odds of clinically significant improvement were higher in exposure if assuming high reliability (OR = 2.16 [1.33–3.49]) but not assuming moderate reliability (OR = 1.64 [0.83–3.28]; Supplementary Table DS6).

**Table 3.** Mean change in internet-delivered exposure therapy versus healthy lifestyle promotion for persistent physical symptoms

Outcome	Scale	Treatment	Change from pre-treatment to post-treatment					Change from pre to 12 months	
			Within-group change		Difference in change (primary endpoint)			Within-group change	
			est (95% CI)	d	est (95% CI)	d	p	est (95% CI)	d
*Primary*									
Somatic symptom burden	PHQ–15	Exposure	−2.7 (−3.5, −1.9)	0.59	−0.7 (−1.8, 0.4)	0.14	0.220	−1.7 (−2.7, −0.7)	0.37
		HLP	−2.0 (−2.8, −1.3)	0.44				–	–
*Secondary*									
Symptom preoccupation	SSD–12	Exposure	−9.5 (−11.3, −7.6)	1.19	−2.7 (−5.3, −0.2)	0.31	0.033	−7.9 (−9.6, −6.1)	0.99
		HLP	−6.7 (−8.5, −5.0)	0.85				–	–
General anxiety	GAD–7	Exposure	−1.4 (−2.4, −0.4)	0.33	−1.2 (−2.6, 0.3)	0.27	0.111	−1.7 (−2.8, −0.6)	0.39
		HLP	−0.3 (−1.3, 0.7)	0.06				–	–
Depression symptoms	PHQ–9	Exposure	−2.4 (−3.3, 1.5)	0.49	−1.3 (−2.6, 0.1)	0.27	0.059	−1.7 (−2.8, −0.6)	0.34
		HLP	−1.1 (−2.0, −0.2)	0.23				–	–
Functional impairment	WD2–12	Exposure	−7.1 (−9.6, −4.6)	0.43	−1.2 (−4.7, 2.3)	0.07	0.495	−7.0 (−10.9, −3.1)	0.43
		HLP	−5.9 (−8.4, −3.5)	0.36				–	–

*Note*: Estimates derived from linear mixed effects regression models fitted on multiply imputed data. All scales were administered as self-report questionnaires via the Internet at the pre-treatment assessment (pre), post-treatment assessment (post), 6 months after the end of treatment, and 12 months after the end of treatment. The PHQ-15 and SSD-12 were also administered each week over the 10-week treatment, thus resulting in 13 measurement points (1 pre, 9 weekly, 1 post, 2 follow-up). For the 12-month follow-up analyses, a spline was added at the post-treatment assessment, to enable modelling of different rates of change for the main and follow-up phases. These models were run on the exposure data only because the healthy lifestyle promotion group was crossed over to exposure therapy after the post-treatment assessment. Standardized within-group effects (ds) were calculated as the negated point estimate for the model-implied change (as listed under ‘est (95% CI)’), divided by the pre-treatment standard deviation for the entire sample in the non-imputed data. Standardized between-group effects (ds) were calculated as the point estimate for the model-implied difference (as listed under ‘est (95% CI)’), divided by the endpoint standard deviation for the entire sample in the non-imputed data. HLP, healthy lifestyle promotion; PHQ-9, Patient Health Questionnaire 9; PHQ-15, Patient Health Questionnaire 15 rephrased to concern the past week only; SSD-12, Somatic Symptom Disorder B-criteria scale 12 rephrased to concern the past week only; WD2-12, 12-item World Health Organization Disability Assessment Schedule 2.0.

### Secondary outcomes: symptom scales, patient satisfaction, and follow-up

As is shown in Table 3, exposure therapy was superior to healthy lifestyle promotion in its average effect on symptom preoccupation (*b* = −2.7 [−5.3 to 0.2]; *p* = 0.033; *d* = 0.31) but not in its effect on general anxiety, depression, or functional impairment. Post-treatment patient satisfaction was significantly higher in exposure therapy (CSQ-8 *M* = 24.1 [*SD* = 4.4, *n* = 74] versus *M* = 20.1 [*SD* = 5.3, *n* = 79]; *p* < 0.001). A qualitative review of free-text responses provided by participants in the HLP group indicated a perceived lack of symptom improvement and suggested that the intervention content was perceived by some as too generic, offering only limited novel information on symptom management. Symptom levels in the exposure condition were largely maintained up to 12 months (Table 3, Supplementary Table DS7).

### Planned moderator analyses

**Figure 2.**
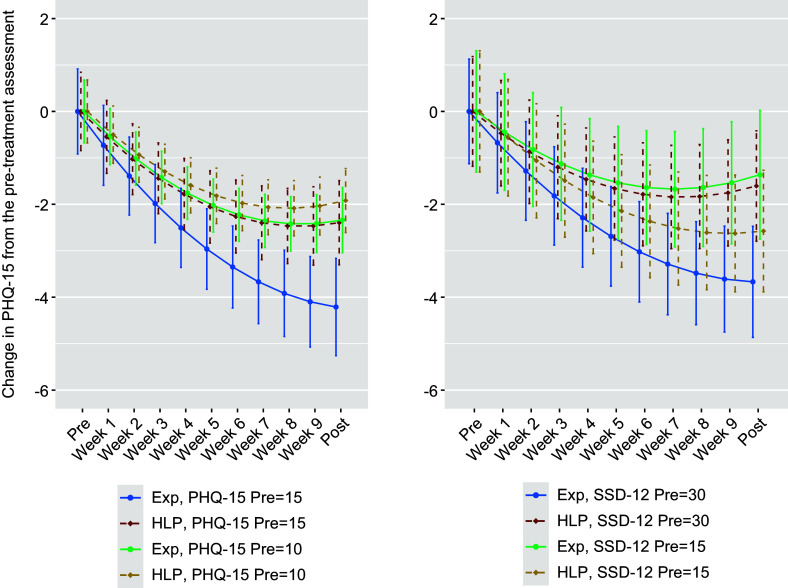
Moderators of the added benefit of exposure therapy versus healthy lifestyle promotion. In these graphs, the reduction in somatic symptom burden (Patient Health Questionnaire; PHQ-15) is plotted as a function of treatment condition (exposure therapy versus healthy lifestyle promotion) and the pre-treatment level of somatic symptom burden (left-hand graph) or pre-treatment symptom preoccupation (right-hand graph; Somatic Symptom Disorder B-criteria scale; SSD-12). The PHQ-15 and SSD-12 were phrased to concern the past week. The predictors were entered as continuous variables, and the particular levels illustrated here, such as a score of 10 versus 15 on the PHQ-15, were chosen for illustrative purposes only. Standardized between-group effects over other levels of each predictor are reported in Supplementary Tables DS9–DS11.

Pre-treatment somatic symptom burden (PHQ-15) and symptom preoccupation (SSD-12) were significant moderators of the difference in mean effect between exposure therapy and healthy lifestyle promotion on somatic symptom burden over the main phase. The effect size was such that a one-point higher pre-treatment PHQ-15 score could be expected to increase the between-group effect by 0.06 standardized *d* units. A one-point higher pre-treatment SSD-12 score could be expected to increase the between-group effect by 0.04 units (see Figure 2).

For the secondary outcome of symptom preoccupation, pre-treatment somatic symptom burden (PHQ-15) and core depression symptoms (the first two items of the PHQ-9) were significant moderators. A one-point higher pre-treatment PHQ-15 score could be expected to increase the between-group effect by 0.08 standardized *d* units. A one-point higher pre-treatment core depression score could be expected to increase the between-group effect by 0.02 units. Neither functional impairment, clinical characteristics related to the type and duration of persistent physical symptoms, sociodemographic variables, adherence, nor recruitment pathway moderated the effect on somatic symptom burden or symptom preoccupation. Details about moderators are reported in Supplementary Tables DS8–DS11.

### Adverse events and deterioration

At least one adverse event – typically a short-term increase in symptoms – was reported by 22% (16/74) of patients in exposure therapy versus 19% (15/79) in healthy lifestyle promotion (OR = 1.10 [0.49–2.47]). One serious event was registered in the exposure condition, where a patient reported having been diagnosed with brain tumors and heart failure during the follow-up period. Minimal clinically important deterioration was reported by 11% (8/74) in exposure versus 9% (7/79) in healthy lifestyle promotion (OR = 1.46 [0.51–4.14]). See Supplementary Tables DS6 and DS12 for details.

## Discussion

This was a randomized controlled trial that compared internet-delivered exposure therapy to internet-delivered healthy lifestyle promotion, delivered over 10 weeks to 161 patients with persistent physical symptoms. The exposure protocol could be tailored for patients suffering from a wide spectrum of physical symptoms and unwanted emotions. In the primary analysis, exposure therapy was not superior to healthy lifestyle promotion in reducing mean subjective somatic symptom burden (*d* = 0.14). Secondary analyses indicated a small but significant advantage in the reduction of symptom preoccupation (*d* = 0.31), but not in the reduction of general anxiety, depression, or functional impairment. Thus, the hypothesis of exposure being superior was corroborated only for the secondary outcome of symptom preoccupation. Symptom levels in the exposure condition were maintained up to twelve months. In planned moderator analyses, a higher pre-treatment somatic symptom burden or symptom preoccupation was predictive of a larger advantage of exposure therapy relative to healthy lifestyle promotion in the reduction of somatic symptom burden. A higher pre-treatment level of somatic symptom burden or depression core symptoms was also predictive of a greater benefit from exposure therapy relative to healthy lifestyle promotion in reducing symptom preoccupation. These moderating effects were moderately sized and in line with a priori hypotheses. Notably, there was no moderating effect of whether the patient’s symptoms were deemed medically unexplained. A strength of this trial was the purely exposure-based treatment protocol, which made between-group differences informative of the benefit of working with exposure and response prevention *specifically*, compared to healthy lifestyle promotion. The treatments were delivered in routine primary care, and broad eligibility criteria also allowed for clinically meaningful moderator analyses.

### Comparison with prior research

Psychological treatments involving exposure have been found to improve health outcomes across a range of conditions, but they can be challenging for both therapists and patients (Pittig, Kotter, & Hoyer, 2019). This was the first trial to evaluate whether there are added benefits of working specifically with exposure and response prevention, compared to a more widely implemented clinical intervention, for patients with a wide range of persistent physical symptoms. The lack of superiority of exposure therapy over healthy lifestyle promotion in the effect on subjective somatic symptom burden (*d* = 0.14) was contrary to our hypothesis. For exposure, a slightly larger mean reduction was seen in the feasibility study (*d* = 0.59 versus *d* = 0.78 based on analogous statistical models) (Hybelius et al., 2022). Potential causes for this discrepancy include random error (*N* = 33 in the feasibility study), the inclusion of participants with primary pathological health anxiety in the feasibility study – a condition that responds well to exposure (Axelsson & Hedman-Lagerlöf, 2019), and that the present study was based in routine care.

The lack of superiority of exposure therapy over healthy lifestyle promotion in reducing somatic symptom burden in this trial (*d* = 0.14) may be contrasted with findings from a recent systematic review and meta-analysis of randomized controlled trials comparing multi-component CBT protocols to rudimentary waitlist, or waitlist-like, control conditions (Hybelius et al., 2024). This review presented a moderate pooled between-group effect on somatic symptom burden (*g* = 0.32 [95% CI, 0.08–0.56]). Against the background of this modest pooled effect versus rudimentary control conditions, the small observed differences between exposure therapy and the relatively stringent control of healthy lifestyle promotion may be less surprising. Further, the observed average benefit of exposure therapy on symptom preoccupation aligns with previous research indicating that treatments involving exposure typically have relatively large within- and between-group effects on this and similar outcomes, such as anxiety about symptoms in asthma and atrial fibrillation (Bonnert et al., 2021; Särnholm et al., 2023). Another example is a recent meta-analysis of psychological interventions for irritable bowel syndrome, which indicated that therapies including exposure are associated with a large average reduction in gastrointestinal-specific anxiety (*g* = 1.05 [95% CI, 0.80–1.31]) (Axelsson et al., 2020, 2023). The superiority observed for the effect on symptom preoccupation thus aligns with the classic view of exposure therapy as a method that primarily targets the emotional component of the patient’s problems, typically reflected in mainstream theoretical models that emphasize the role of anxiety or fear in relation to avoidance behaviors (Benito et al., 2024; Vlaeyen & Linton, 2000; Warwick & Salkovskis, 1990).

This trial identified effect moderators in terms of higher levels of pre-treatment somatic symptom burden, symptom preoccupation, and depressive symptoms being predictive of a greater advantage of exposure therapy over healthy lifestyle promotion. For example, on average, a patient with a PHQ-15 score of 10 could expect a near-zero added effect of exposure therapy on somatic symptom burden (*d* = 0.08) and a small added effect on symptom preoccupation (*d* = 0.31), whereas a patient with a PHQ-15 score of 15 could expect a small to moderate added effect on somatic symptom burden (*d* = 0.37) and a moderate added effect on symptom preoccupation (*d* = 0.62). These moderating effects on the PHQ-15 were in line with a priori hypotheses and indicate that it is possible to identify patients who benefit more from exposure therapy than from healthy lifestyle promotion. This is notable in light of the broader literature on persistent physical symptoms, plagued by conflicting diagnostic categories that often lack clear clinical utility (Kohlmann, Löwe, & Shedden-Mora, 2018).

### Recruitment and implementation in the primary care setting

This trial gave valuable insights into the challenges of recruiting patients with persistent physical symptoms for exposure therapy in routine primary care. While we first intended for patients to be referred primarily via the general practitioners at the clinic, we had to adopt a more pragmatic approach to recruitment. Several factors likely contributed to this. Based on open discussions and brief written input from eight general practitioners, recurrent concerns included time constraints, unfamiliarity with considering distress related to persistent physical symptoms as a transdiagnostic phenomenon, and with referring these patients to a mental health professional. The latter aligns with previous research suggesting that general practitioners often perceive that patients who seek a physical explanation for their symptoms may not be open to behavioral interventions (Hanssen, Ras, & Rosmalen, 2021). Similar to previous authors, we suspect that improving practitioners’ knowledge about biopsychosocial perspectives on persistent physical symptoms could aid dissemination (Kitselaar et al., 2021; Lehmann et al., 2021; Toussaint et al., 2025).

### Limitations

This study had several limitations. Because there was no passive control group, it is not possible to determine to what degree patients would have improved spontaneously. On the other hand, in the current sample, participants had been bothered by physical symptoms for a mean of 12 years, and the natural course for this patient population in European primary care is typically relatively stable (Kustra-Mulder, Löwe, & Weigel, 2023). Furthermore, with few exceptions, other structured psychological treatments have been found superior to waitlists (e.g., Hybelius et al., 2024). The cross-over design did not permit long-term comparisons, but offered ethical advantages. Another characteristic of the trial that may be regarded as a limitation for demonstrating superiority was the relatively broad eligibility criteria. Because patients could score as low as 2 out of 30 on the primary outcome, there was sometimes little room for improvement. On the other hand, this design choice was also a key strength of the trial, as it allowed us to empirically examine who benefits more from exposure compared to healthy lifestyle promotion, rather than merely assuming this based on limited prior evidence. Had we instead recruited only participants with high levels of somatic symptom burden or symptom preoccupation, the moderating effect of these variables would have been weaker (Bland & Altman, 2011). Furthermore, the shift from recruitment solely through primary care to additionally include self-referred participants may have affected the composition of the sample. Notably, however, recruitment path did not significantly moderate the treatment effect. Concerning the measurement strategy, the 1-week time frame for the PHQ-15 is not widely adopted and implies a lack of reference data. That said, Cronbach’s alpha was 0.84 for the one-week version in a previous study (Joustra, Janssens, Schenk, & Rosmalen, 2018), and we replicated that the measurement properties of this version appear promising in a recently submitted secondary study. Furthermore, there were no objective process measures, such as directly observed behavior during exposure exercises, or physical activity measured using wristband accelerometers. Because the same therapists administered both treatments, contamination cannot be entirely ruled out. On the other hand, both interventions were highly structured, and the information necessary for behavior change was conveyed via the standardized treatment text modules.

### Clinical implications and recommendations for future research

Exposure therapy can be demanding, and the population of patients bothered by persistent physical symptoms is large and heterogeneous. To prevent overtreatment and facilitate personalized care, it is crucial to distinguish between highly functioning individuals with mild problems and people experiencing pronounced symptom burden and distress. From an ethical and utility perspective, exposure therapy should be aimed toward the latter group. Our findings suggest that patients need to score approximately 15 or more on the PHQ-15, or approximately 25 or more on the SSD-12, to expect a clear benefit from exposure over the lower threshold intervention of healthy lifestyle promotion. If no distinction is made between high and low scorers on somatic symptom burden or symptom preoccupation, the average benefit of exposure therapy over the arguably more intuitive approach of healthy lifestyle promotion appears to be small on average. For subgroups with milder problems, healthy lifestyle promotion may serve as a resource-efficient intervention, aligning with a stepped care approach to the management of persistent physical symptoms (Toussaint et al., 2025).

Future publications are planned on cost-effectiveness, the role of basic emotions, and potential effect mediators and process variables responsible for treatment effects. Future research could also explore efficient ways of integrating exposure strategies into care pathways for patients experiencing pronounced distress related to persistent physical symptoms in routine care, perhaps as an additive component to healthy lifestyle promotion. Studies may model the optimal combination of treatment components to include alongside exposure to build an effective multicomponent protocol, and how the effects of exposure-based therapies differ based on whether these are transdiagnostic or developed with a specific focus on one diagnosis or symptom domain.

### Conclusion

Compared with healthy lifestyle promotion, in this mixed persistent symptom population, exposure therapy does not appear to show a notable average benefit except for a small advantage on symptom preoccupation. Based on moderator analyses, substantial benefits are seen when patients have a PHQ-15 score of approximately 15 or more or an SSD-12 score of approximately 25 or more.

## Supporting information

Hybelius et al. supplementary material 1 Hybelius et al. supplementary material

Hybelius et al. supplementary material 2 Hybelius et al. supplementary material

## Supplementary material

To view supplementary material for this article, please visit http://doi.org/10.1017/S0033291725101244.
