# Supplementary material for: Effect of internet-delivered exposure therapy versus healthy lifestyle promotion for patients with persistent physical symptoms (SOMEX1): a randomized controlled trial with planned moderator analysis
Source: Psychol Med. 2025 Aug 8;55:e226. doi: 10.1017/S0033291725101244 (PMC12360694; doi:10.1017/S0033291725101244)
Supplement: Hybelius et al. supplementary material 1 — Hybelius et al. supplementary material [file S0033291725101244sup001.pdf]

Supplement for:

Effect of internet-delivered exposure therapy versus healthy lifestyle promotion for patients with persistent physical symptoms (SOMEX1):
A randomized controlled trial with planned moderator analysis

Figure DS1. Schematic overview of trial design 2

Table DS1. Elaboration on eligibility criteria 3

Table DS2. Overview of therapist characteristics. 5

Table DS3. Overview of treatment content 6

Figure DS2. Model presented in exposure therapy for persistent physical symptoms. 12

Supplementary Methods 13

Table DS4. Exploratory secondary analyses of subdomains and subgroups pertaining to mean change in Internet-delivered exposure therapy vs. standardized healthy lifestyle promotion for distress related to persistent physical symptoms 14

Table DS5. Exploratory secondary analyses of specific lifetime diagnostic groups pertaining to mean change in Internet-delivered exposure therapy vs. standardized healthy lifestyle promotion for distress related to persistent physical symptoms 15

Table DS6. Key dichotomous outcomes in Internet-delivered exposure therapy vs. standardized healthy lifestyle promotion for distress related to persistent physical symptoms 17

Table DS7. Mean scores in Internet-delivered exposure therapy vs. standardized healthy lifestyle promotion for distress related to persistent physical symptoms 18

Table DS8. Moderator analyses pertaining to mean change in exposure therapy vs. standardized healthy lifestyle promotion 19

Table DS9. Estimated treatment difference in mean change as a function of significant moderators 21

Table DS10. Estimated treatment difference in mean change in the Patient Health Questionnaire 15 as a function of moderators in a non-planned multivariable model 22

Table DS11. Estimated treatment difference in mean change in the Somatic Symptom Disorder B-criteria scale 12 as a function of moderators in a non-planned multivariable model 23

Table DS12 Adverse events as reported in the post-treatment assessment. 24

Table DS13 Test of potential gender effect on exclusions due to primary psychiatric disorder 25

References 26

Figure DS1. Schematic overview of trial design

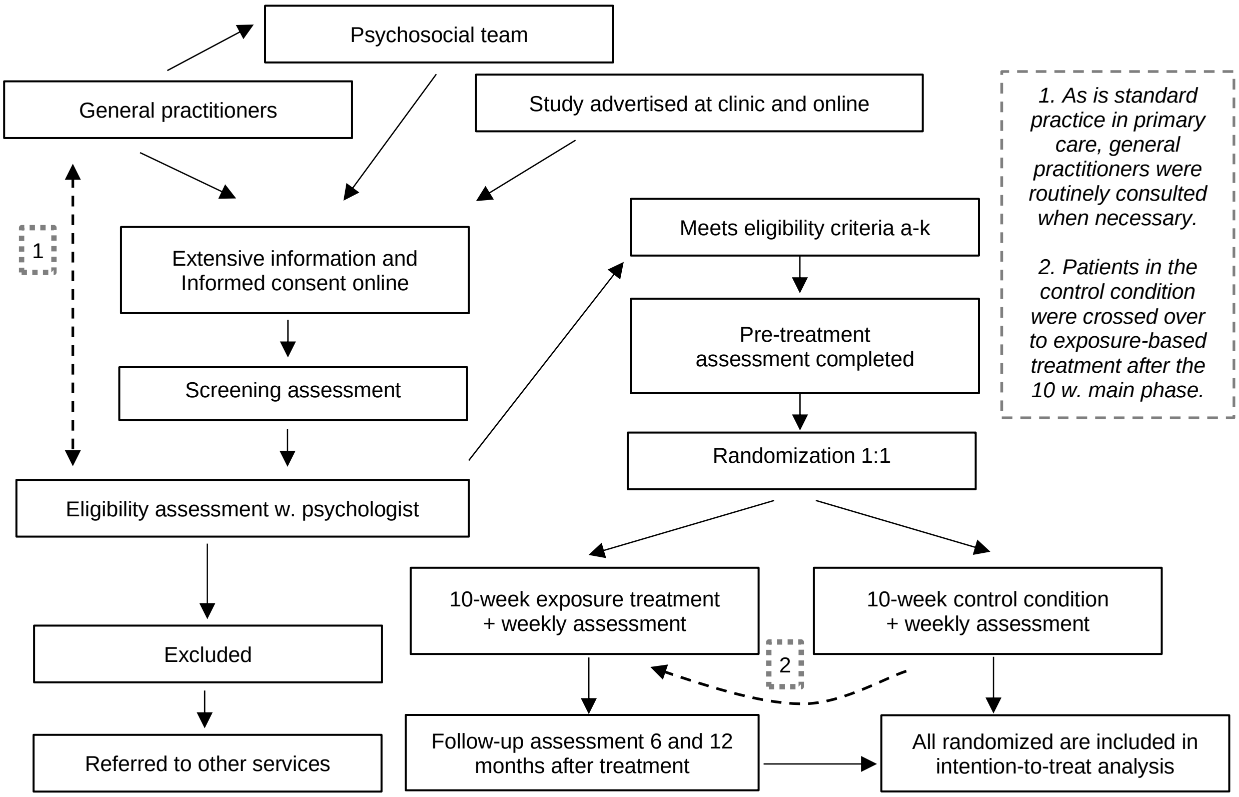

Table DS1. Elaboration on eligibility criteria

	Criterion	Method of assessment	Note
**a**	(i) Either much bothered by at least one somatic symptom (2 points on at least one item of the PHQ-15) or at least a moderate overall somatic symptom burden (PHQ-15 sum ≥10), with (ii) recurrent distress related to somatic symptoms ≥4 months	Part of application/screening	The being “bothered a lot” by at least one symptom criterion was derived from previous work (Claassen-van Dessel et al. 2018; Häuser et al. 2015). The cut-off of 10 is widespread (Bailer et al. 2016; Körber et al. 2011) and has been argued to stand for a moderate symptom burden (Kroenke et al. 2002).
**b**	Symptoms not best explained by, and clinical picture not dominated by, severe health anxiety or a non-somatoform psychiatric disorder such as depression, panic disorder, primary insomnia, a chronic stress disorder, or an acute stress disorder	Psychiatric interview aided by the Mini-International Neuropsychiatric Interview (MINI) (Sheehan et al. 1998) and the Health Preoccupation Diagnostic Interview (HPDI) (Axelsson et al. 2016)	Comorbidities were allowed, but patients were referred to routine clinical services for mood, anxiety, and stress disorders that were clearly the primary condition.
**c**	Interested in completing an intense psychological treatment with the aim of reducing distress related to somatic symptoms	Psychiatric interview	The interviewer made clear that participants were expected to work with the treatment on a daily basis.
**d**	Adult (≥18 years old)	Part of application/screening: Secure electronic identification was required to register for a personal account on the web platform, and this identification was tied to the applicant’s personal identity number (Ludvigsson et al. 2009)	
**e**	Living in Stockholm County	Part of application/screening: Secure electronic identification tied to the applicant’s personal identity number (Ludvigsson et al. 2009), which in turn was tied to home address in the medical records system	The primary care clinic had Stockholm as its catchment area.
**f**	Can read and write in Swedish	Part of application/screening, and was also discussed as part of the psychiatric interview	

**g**	Not severe psychiatric disorder or suicidal ideation	Part of application/screening, and was also assessed as part of the psychiatric interview which was aided by the MINI and HPDI. The assessment of mood disorders followed DSM criteria.	The primary care clinic was tasked with managing mild to moderate psychiatric disorders only. Examples of severe conditions are severe depressive disorder or psychotic disorder.
**h**	No clear medical risk in taking part in exposure-based treatment (e.g., pregnancy) and somatic condition (e.g., recent cancer diagnosis), or treatment for somatic condition (e.g., recent chemotherapy), does not make the treatment unfeasible	Part of application/screening, and was also discussed as part of the psychiatric interview	One common concern was if it would be appropriate for the applicant to engage in intense physical activity. Another concern was other therapies that could be demanding to combine with the present trial.
**i**	Continuous psychotropic medication either non-existent or stable in past 4 weeks, and expected to remain stable over the main phase of the trial	Part of application/screening, and was also discussed as part of the psychiatric interview; focusing on antidepressants, anticonvulsants, mood stabilizers, and antipsychotics.	This was intended to be similar to the routine care environment in that prior to psychotherapy, typically, medications are expected to be reasonably stable.
**j**	Not severe alcohol or substance use disorder likely to interfere with treatment	Part of application/screening, which included the Alcohol Use Disorders Identification Test (AUDIT) (Saunders et al. 1993) and the Drug Use Disorders Identification Test (DUDIT) (Berman et al. 2005). Also assessed during the psychiatric interview which was aided by the MINI.	An alcohol or substance use disorder was not a reason for exclusion per se. This criterion was only concerned with cases where problems were so severe that treatment would not be feasible.
**k**	Not planned absence for more than 1 week during the intended main phase	Part of application/screening, and was also discussed as part of the psychiatric interview	If possible, participation was postponed to a later date (cohort).
**l**	Complete pre-treatment assessment	Applicants who met all other criteria were scheduled for a start week (cohort), usually within a few weeks from the eligibility interview. Ca 4 days prior to the start of that week, applicants received a link to the online assessment via SMS.	Applicants had ca 4 days to complete the assessment. Those who did not do so within ca 2 days were contacted and received reminders via SMS, telephone, and e-mail.

Note. PHQ-15, Patient Health Questionnaire 15.

Table DS2. Overview of therapist characteristics.

		Patients treated		Prior experience in no. of years	
**Therapist**	**Profession**	**Exp**	**HLP**	**Psychotherapy**	**Working with PPS**
A	Psychologist	39	40	1.5	0.5
B	Psychologist	10	11	4.5	0
C	Psychotherapist	9	9	8	0
D	Psychologist	7	6	0.5	0
E	Psychologist	5	5	0.5	0
F	Psychologist	5	5	1	0
G	Psychologist	4	4	8	8
H	Psychologist	2	2	8	8
I	Psychologist	0	1	1.5	1

Notes. The total number of patients randomized and treated in this trial was *N* = 161. The same patient was sometimes treated by more than one therapist, which is why the total number of patients reported above exceeds 161. Exp, Internet-delivered exposure therapy; HLP, Internet-delivered healthy lifestyle promotion; PPS, persistent physical symptoms.

Table DS3. Overview of treatment content

Module	Exposure therapy	Healthy lifestyle promotion
1	*Introduction to tailored exposure treatment for persistent physical symptoms*	*Introduction to healthy lifestyle promotion for management of psychological distress and physical symptoms*
	Overview of treatment content and the internet-based format. Psychoeducation about the role of psychological factors in the maintenance and worsening of physical symptoms and functional impairment. Introduction of three fictitious cases illustrating the exposure-based treatment model (see Figure DS2) across different symptom domains (pain and comorbid fatigue, cardiopulmonary symptoms, gastrointestinal complaints). Introduction of a behavior diary for self-monitoring of reactions to, and use of, behavioral strategies aimed at short-term relief of physical symptoms, to increase awareness of areas for exposure and response prevention later in treatment. These strategies are referred to as "symptom behaviors" and include seeking symptom-related reassurance or information, excessive monitoring and checking of bodily sensations and symptoms, safety behaviors intended to increase the sense of security or preparedness to manage physical symptoms should they occur (e.g., checking where the nearest bathroom is located or planning one's day to minimize physical symptoms), and avoidance behaviors. Introduction of mindful observation of bodily distress as an alternative approach to engaging in symptom behaviors.	Overview of treatment content and the internet-based format. A symptom diary is introduced for self-monitoring of physical symptoms, strategies used to manage symptoms, and engagement in health-related behaviors (i.e., physical activity, diet, sleep strategies, strategies to support mental wellbeing, as well as any noteworthy or distressing events during the day). The text emphasizes that improved lifestyle behaviors support both physical and mental health, including persistent physical symptoms and related distress. The rationale for self-monitoring is to identify patterns in symptom fluctuations and behavioral habits that positively influence well-being. Patients are also encouraged to begin drafting a structured health plan, focusing on lifestyle habits they wish to improve. Principles for effective goal setting are provided, and patients are encouraged to revisit and revise their goals throughout treatment.
	Suggested homework:<br>Daily logging in the behavior diary.<br>Practice replacing a frequently used symptom behavior with mindful observation of bodily distress.	*Suggested homework:*<br>Daily logging in the symptom diary.<br>Goal setting (health plan).
2	*Introduction to exposure and response prevention*	*Physical activity*
	Introduction of exposure based on a habituation rationale, also framed as a way to learn new strategies for managing symptoms and related distress, adjusting unhelpful expectations, and increasing long-term well-being. Interoceptive exposure is	Patients are provided with education on the positive health effects of physical activity. Guidelines outlining targets for weekly moderate- and vigorous-intensity physical activity are included. Patients are referred to the national 1177.se service for additional

	introduced (i.e., actively provoking unpleasant physical sensations to achieve long-term reduction of unwanted emotional responses to those sensations). Patients are provided with a list of pre-specified interoceptive exposure exercises and are encouraged to engage in these until habituation or a marked reduction in emotional reactivity has occurred. Furthermore, patients receive a more detailed rationale for response prevention, based on the previously introduced treatment model and insights gained from the behavior diary. Tools for formulating realistic and measurable goals for response prevention across all previously described domains of symptom behaviors are provided. Patients are encouraged to revisit and revise their goals throughout the treatment.	advice on starting a new exercise routine, inspiration on specific sports and activities, and information on how to apply for physical activity on prescription.
	Suggested homework:<br>Behavior diary.<br>Interoceptive exposure.<br>Goal formulation for response prevention.<br>Response prevention.	*Suggested homework:*<br>Symptom diary.<br>Continued engagement in daily healthy lifestyle habits according to the health plan.
**3**	*Tailored exposure exercises*	*Healthy diet, alcohol and tobacco habits*
	The rationale for exposure is reviewed and further elaborated, now also incorporating tailored exposure exercises in real-world situations. Patients are given instructions for planning and performing daily exposure exercises tailored to their specific symptoms and treatment goals. Key principles for effective exposure are presented, emphasizing that exposure should ideally be structured, sufficiently challenging, frequently repeated, and conducted without the use of behavioral strategies aimed at short-term relief of symptoms or related distress.	Patients are provided with education on the positive health effects of a healthy and balanced diet. Dietary guidelines and advice on beneficial eating habits are included. The risks and harmful effects of alcohol and tobacco use are also addressed. Patients are referred to the national 1177.se service for further guidance on improving dietary and alcohol habits. Additionally, support for smoking cessation and referrals to services that assist with quitting tobacco use are provided.
	Suggested homework:<br>Response prevention.<br>Interoceptive exposure (if still relevant).<br>Tailored exposure in vivo.	*Suggested homework:*<br>Symptom diary.<br>Continued engagement in daily healthy lifestyle habits according to the health plan.

4	*Managing worry and rumination, introduction to imaginal exposure*	*Management of low mood, worry, and anxiety*
	The module begins with additional guidance and a review of key principles for successful exposure.<br>Psychoeducation on catastrophic thoughts, worry, and rumination builds on earlier information about the long-term negative consequences of behavioral strategies aimed at short-term relief of symptoms and related distress. This is achieved by highlighting the problematic long-term outcomes of avoiding catastrophic thoughts and by conceptualizing worry and rumination as covert symptom behaviors.<br>Imaginal exposure is introduced as a strategy for confronting catastrophic thoughts. Patients are encouraged to write an “illness-story”; a detailed written narrative describing the scenario underlying their recurrent catastrophic thoughts related to physical symptoms (e.g., “my pain will become so debilitating that I will end up unable to walk”) or fear of serious illness (often centered around receiving a diagnosis such as cancer or a severe neurological disorder). Patients are encouraged to repeatedly read or listen to a recording of the illness-story until habituation occurs.	Patients are provided with psychoeducation covering the differences between normal fluctuations in mental wellbeing and persistent, pronounced symptoms of depression or anxiety that warrant medical attention. Advice on managing low mood, worry, and anxiety is provided, including recommendations for physical activity (due to its antidepressant and anxiolytic effects), relaxation techniques, and mindfulness. Patients are referred to the national 1177.se service for further guidance on improving mental health, seeking further professional support if needed, and accessing various relaxation and mindfulness exercises.
	Suggested homework:<br>Continued engagement in daily, tailored exposure exercises in various forms.<br>Response prevention.<br>Imaginal exposure.	*Suggested homework:*<br>Symptom diary.<br>Continued engagement in daily healthy lifestyle habits according to the health plan.
5	*Plan for further improvement and healthcare utilization.*	*Further lifestyle habits for a balanced life – stress management, sleeping habits, healthy relationships, and healthcare utilization*
	Repetition of principles for effective exposure and response prevention. Elaboration on common pitfalls in exposure therapy (e.g., finding time, planning ahead, performing daily exposure exercises regardless of mood) and strategies to address these. For patients who found imaginal exposure relevant (i.e., experienced distress when exposing themselves to their illness-story), guidance	Patients are provided with education on the importance and positive health effects of balancing activity and recovery, along with psychoeducation about the key differences between transient stress and chronic, unhealthy stress. The negative health effects of chronic stress are explained. The module also offers advice on sleep hygiene, the importance of healthy relationships,

	is provided on how to elaborate the story, and, if necessary, incorporate fear of death.<br>For patients who perceive that they engage in excessive healthcare seeking or, conversely, avoid necessary healthcare consultations, guidance for functional healthcare use and tools for formulating a plan for future consultations are provided.	ways to overcome involuntary loneliness, and brief guidance on seeking further medical care if needed. Patients are referred to the national 1177.se service for further advice and guidance on stress management, sleep habits, and the significance of close, healthy relationships.
	Suggested homework:<br>Various forms of tailored exposure. Response prevention.<br>Writing a structured long-term plan for future healthcare seeking.	*Suggested homework:*<br>Symptom diary.<br>Continued engagement in daily healthy lifestyle habits according to the health plan.
**6**	*Continued exposure and response prevention*	*Continued monitoring of symptoms and adherence to the health plan*
	Repetition of principles for effective exposure and response prevention. Patients are encouraged to continue and expand their daily work with exposure and response prevention.	Repetition of principles for effective goal setting. Patients are encouraged to continue and expand their engagement in healthy lifestyle habits. Declines in motivation and resistance to change are normalized.
	Suggested homework:<br>Continued engagement in daily exposure exercises and response prevention.	*Suggested homework:*<br>Symptom diary.<br>Continued engagement in daily healthy lifestyle habits according to the health plan.
**7**	*Continued exposure and response prevention*	*Continued monitoring of symptoms and adherence to the health plan*
	Repetition of the principles for effective exposure and response prevention. Patients are encouraged to continue and expand their daily exposure and response prevention work.<br>A rationale is provided for overlearning by approaching the situations and experiences most feared by the patient.	Repetition of principles for effective goal setting. Patients are encouraged to continue and expand their engagement in healthy lifestyle habits. Declines in motivation and resistance to change are normalized.
	Suggested homework:<br>Various forms of tailored exposure. Response prevention.<br>Overlearning by engaging in more challenging exposure exercises.	*Suggested homework:*<br>Symptom diary.<br>Continued engagement in daily healthy lifestyle habits according to the health plan.
**8**	*Continued exposure and response prevention*	*Continued monitoring of symptoms and adherence to the health plan*

	Repetition of the principles for effective exposure and response prevention. Patients are encouraged to continue and expand their daily exposure and response prevention work.	Repetition of principles for effective goal setting. Patients are encouraged to continue and expand their engagement in healthy lifestyle habits. Declines in motivation and resistance to change are normalized.
	Suggested homework:<br>Continued engagement in daily exposure exercises and response prevention.	*Suggested homework:*<br>Symptom diary.<br>Continued engagement in daily healthy lifestyle habits according to the health plan.
**9**	*Continued exposure and response prevention*	*Continued monitoring of symptoms and adherence to the health plan*
	Repetition of the principles for effective exposure and response prevention. Patients are encouraged to continue and expand their daily exposure and response prevention work.	Repetition of principles for effective goal setting. Patients are encouraged to continue and expand their engagement in healthy lifestyle habits. Declines in motivation and resistance to change are normalized.
	Suggested homework:<br>Continued engagement in daily exposure exercises and response prevention.	*Suggested homework:*<br>Symptom diary.<br>Continued engagement in daily healthy lifestyle habits according to the health plan.
**10**	*Continued improvement and relapse prevention*	*Continued monitoring of symptoms and adherence to the health plan*
	Advice on continued exposure and a summary of the treatment principles. Patients are encouraged to continue expanding their work with exposure and response prevention after completing treatment.<br>Psychoeducation is provided on principles for maintaining treatment gains, supporting continued improvement, and preventing relapse. Patients are encouraged to save the treatment materials and to formulate a structured plan for relapse prevention.	Repetition of principles for effective goal setting. Patients are encouraged to continue and expand their engagement in healthy lifestyle habits. Declines in motivation and resistance to change are normalized.
	Suggested homework:	*Suggested homework:*

	Continued engagement in daily exposure exercises and response prevention.<br>Writing a structured long-term plan for continued exposure and relapse prevention.	Symptom diary.<br>Continued engagement in daily healthy lifestyle habits according to the health plan.<br>Writing a summary of key insights from working with healthy lifestyle promotion.

Note. Both therapies were delivered via the same web platform, “1177 Stöd- och behandling”, which is the most widespread platform for text-based psychological online treatment in Sweden.

Figure DS2. Model presented in exposure therapy for persistent physical symptoms.

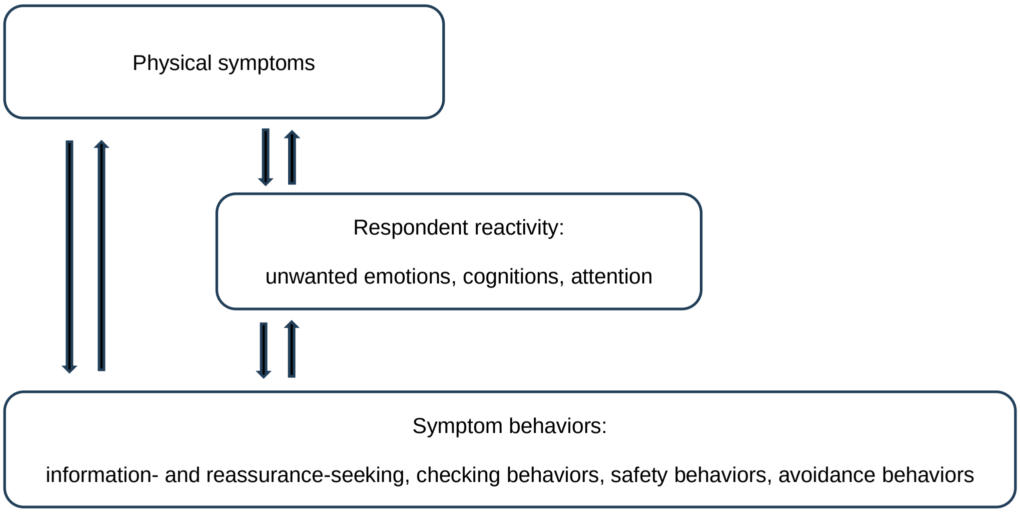

Note. See Table DS3 for a description of the processes in the model.

Supplementary Methods

Details pertaining to recruitment – justification for primary eligibility criterion

This trial aimed to recruit a heterogeneous sample with persistent physical symptoms. We did not focus on any specific diagnosis due to the difficulty of establishing the diagnosis of somatic symptom disorder in the feasibility trial (Hybelius et al. 2022) and the realization that current diagnostic approaches remain contested (Kohlmann et al. 2018) while little is known about how to delineate a subgroup with persistent physical symptoms that is likely to respond to exposure therapy. Participants had to be either much bothered by at least one physical symptom [i.e., scoring 2 points on at least one item of the Patient Health Questionnaire 15 (PHQ-15) (Claassen-van Dessel et al. 2018; Häuser et al. 2015), or report at least a moderate overall somatic symptom burden [i.e., a PHQ-15 sum of ≥ 10 (Bailer et al. 2016; Körber et al. 2011), with a duration of at least 4 months. Physical symptoms were not required to be medically unexplained, could belong to any symptom domain (e.g., pain, gastrointestinal, cardiopulmonary, fatigue), and be associated with any combination of unwanted emotions (e.g. anger, anxiety, fear, shame).

Details pertaining to the statistical analysis

Coding of the variable 'medically unexplained symptoms': In this clinical trial, the focus was not on whether the participants' symptoms were to be regarded as medically unexplained or not. Attempts to make this distinction, however, have a long history in this research field. With full knowledge of recognized threats to the reliability of this approach, we wanted to assess its merits on empirical grounds. Clinicians conducting the eligibility interview were therefore instructed to, to the best of their abilities, classify each participant's symptoms as being either medically explained or unexplained (binary variable). Participants were considered to report medically unexplained symptoms if none of the presenting somatic symptoms, i.e., symptoms that the patient wanted to address in treatment, appeared to be clearly related to a diagnosed somatic condition. For the purpose of this analysis, based on the most widespread practice of the field (Burton et al. 2020; Smith and Dwamena 2007), symptoms part of a functional somatic syndrome, such as irritable bowel syndrome and fibromyalgia, were classified as medically unexplained.

Completion of treatment was defined as having initiated at least five out of ten modules in both exposure therapy and healthy lifestyle promotion. Additionally, analyses concerning this variable were repeated utilizing more conservative criteria for treatment completion in the exposure condition (initiated ≥ 5 modules and performed ≥ 3 exposure exercises).

Response to treatment was estimated based on the criterion for clinically significant improvement (CSI) (Jacobson and Truax 1991) for patients scoring 10 or higher on the PHQ-15 before treatment, as the CSI requires a clinical pre-treatment score to be relevant. Analyses were performed both assuming a high ($r = 0.93$) (Liao et al. 2016) and moderate ($r = 0.65$) (Han et al. 2009) test-retest reliability on the PHQ-15. Response to treatment for the full sample was based on the minimal clinically important difference (MID; a decrease of at least 3 in the PHQ-15 sum score for the individual patient) (Hybelius et al. 2024). Further, *unwanted outcomes* were conceptualized as the minimal clinically important deterioration (an increase of 3 or more on the PHQ-15) and also as reporting of one or more adverse events in the post-treatment assessment.

Missing data was multiply imputed using chained equations in mice 3.16.0 (van Buuren and Groothuis-Oudshoorn 2011). We imputed 100 datasets with 20 iterations per imputation using predictive mean matching, separately for each treatment to maintain interaction effects.

For the follow-up analyses (6 and 12 months) of the primary and secondary outcomes, a spline was added at the post-treatment assessment. This was done to enable modelling of different rates of change for the main and follow-up phases. These models were run on the exposure data only as the healthy lifestyle promotion group was crossed over to exposure therapy after the post-treatment assessment.

Table DS4. Exploratory secondary analyses of subdomains and subgroups pertaining to mean change in Internet-delivered exposure therapy vs. standardized healthy lifestyle promotion for distress related to persistent physical symptoms

**Outcome**	**Treatment**	**Means and SDs based on observed data**						**Change from pre-treatment to post-treatment**			
								**Within-group change**		**Difference in change**	
		**Pre-treatment**			**Post-treatment**			**est (95% CI)**	***d***	**est (95% CI)**	***d***
		M	***SD***	***n***	***M***	***SD***	***n***				
Subdomains of symptom burden											
Cardiopulmonary symptoms	Exp	0.5	0.5	80	0.3	0.4	80	-0.2 (-0.2, -0.1)	0.39	-0.1 (-0.2, 0.03)	0.18
	HLP	0.5	0.4	81	0.4	0.4	81	-0.1 (-0.2, -0.02)	0.22		
Fatigue symptoms	Exp	1.2	0.6	80	0.8	0.7	80	-0.3 (-0.4, -0.2)	0.50	-0.1 (-0.3, 0.03)	0.20
	HLP	1.3	0.6	81	1.1	0.6	81	-0.2 (-0.3, -0.1)	0.28		
Gastrointestinal symptoms	Exp	1.2	0.6	80	0.8	0.7	80	-0.2 (-0.3, -0.1)	0.31	-0.04 (-0.2, 0.1)	0.06
	HLP	1.3	0.6	81	1.1	0.6	81	-0.2 (-0.3, -0.1)	0.26		
Pain symptoms	Exp	1.0	0.5	80	0.7	0.5	80	-0.2 (-0.3, -0.1)	0.39	-0.01 (-0.1, 0.1)	0.02
	HLP	1.0	0.5	81	0.8	0.5	81	-0.2 (-0.3, -0.1)	0.38		
Pre-treatment score of at least 1											
Cardiopulmonary symptoms	Exp	1.3	0.3	46	0.9	0.4	42	-0.5 (-0.7, -0.4)	1.28	-0.4 (-0.6, -0.1)	0.92
	HLP	1.3	0.3	53	1.0	0.5	53	-0.2 (-0.4, 0.01)	0.43		
Fatigue symptoms	Exp	1.5	0.4	56	1.0	0.6	51	-0.4 (-0.5, -0.3)	0.64	-0.2 (-0.4, 0.02)	0.25
	HLP	1.5	0.4	61	1.2	0.6	60	-0.2 (-0.4, -0.1)	0.37		
Gastrointestinal symptoms	Exp	1.5	0.4	50	0.9	0.5	45	-0.3 (-0.5, -0.2)	0.52	-0.1 (-0.3, 0.1)	0.17
	HLP	1.5	0.4	51	1.2	0.6	49	-0.2 (-0.4, -0.1)	0.24		
Pain symptoms	Exp	1.3	0.3	46	0.9	0.4	42	-0.3 (-0.4, -0.2)	0.65	-0.1 (-0.2, 0.1)	0.16
	HLP	1.3	0.3	53	1.0	0.5	53	-0.2 (-0.3, -0.1)	0.49		

Notes. Scores represent PHQ-15 subscales; each with a theoretical range of 0 to 2. Estimates derived from linear mixed effects regression models fitted on multiply imputed data. All scales were administered as self-report questionnaires via the Internet at the pre-treatment assessment (pre), each week over the 10-week treatment, at post-treatment assessment (post), and 6 and 12 months after the end of treatment (not reported here). Standardized within-group effects (*d*s) were calculated as the negated point estimate for the model-implied change (as listed under "est (95% CI)"), divided by the pre-treatment standard deviation for the entire sample in the non-imputed data. Standardized between-group effects (ds) were calculated as the point estimate for the model-implied difference (as listed under "est (95% CI)"), divided by the endpoint standard deviation for the entire sample in the non-imputed data. Exp, Internet-delivered exposure therapy; HLP, Internet-delivered healthy lifestyle promotion; PHQ-15, Patient Health Questionnaire 15 rephrased to concern the past week only.

Table DS5. Exploratory secondary analyses of specific lifetime diagnostic groups pertaining to mean change in Internet-delivered exposure therapy vs. standardized healthy lifestyle promotion for distress related to persistent physical symptoms

		Somatic symptom burden: Patient Health Questionnaire 15 (PHQ-15)							Symptom preoccupation: Somatic Symptom Disorder B-criteria scale 12 (SSD-12)						
**Diagnosis**	**Tx**	**Observed data**			**Estimates from linear mixed effects regression**				**Observed data**			**Estimates from linear mixed effects regression**			
		**Pre-treatment**			**Pre to post change**		**Tx difference in change**		**Pre-treatment**			**Pre to post change**		**Tx difference in change**	
		M	***SD***	***n***	**est**	***d***	**est (95% CI)**	***d***	***M***	***SD***	***n***	**est**	***d***	**est (95% CI)**	***d***
Asthma	Exp	12.1	5.0	19	-2.5	0.55	-0.2 (-2.3, 2.0)	0.04	23.2	7.7	19	-10.3	1.29	-0.5 (-6.2, 5.1)	0.06
n = 35/161 (22%)	HLP	11.1	4.2	16	-2.3	0.51			24.3	7.8	16	-9.7	1.22		
Atopic dermatitis	Exp	9.6	3.8	8	-2.7	0.59	-0.2 (-2.9, 2.5)	0.05	25.3	6.9	17	-9.2	1.16	0.2 (-6.1, 6.4)	-0.02
n = 25/161 (16%)	HLP	12.1	4.5	8	-2.5	0.54			23.1	9.8	8	-9.4	1.18		
Cancer (any)	Exp	10.9	7.5	9	-3.5	0.77	-2.0 (-4.1, 0.2)	0.40	26.0	9.8	9	-12.8	1.61	-5.8 (-11.2, -0.4)	0.66
n = 21/161 (13%)	HLP	11.2	4.4	12	-1.6	0.34			22.8	7.3	12	-6.9	0.87		
Diabetes	Exp	13.0	4.6	3	-3.0	0.65	-0.4 (-7.1, 6.2)	0.09	23.3	4.5	3	-8.6	1.08	-3.1 (-15.9, 9.8)	0.34
n = 7/161 (4%)	HLP	9.5	4.8	4	-2.6	0.56			20.0	10.1	4	-5.5	0.70		
Exhaustion disorder	Exp	12.2	4.6	24	-2.6	0.56	-0.5 (-2.3, 1.2)	0.11	24.4	9.3	24	-8.8	1.11	-3.3 (-6.8, 0.2)	0.37
n = 54/161 (34%)	HLP	13.3	4.5	30	-2.1	0.45			22.1	7.8	30	-5.5	0.70		
Fibromyalgia	Exp	14.5	4.3	16	-3.5	0.75	-1.1 (-4.0, 1.8)	0.22	22.1	6.7	16	-10.5	1.32	-8.6 (-14.5, -2.7)	0.97
n = 22/161 (14%)	HLP	15.3	4.1	6	-2.4	0.51			19.8	4.3	6	-1.9	0.24		
Hiatal hernia	Exp	12.2	2.9	10	-3.0	0.65	-1.4 (-3.8, 1.1)	0.28	23.4	5.2	10	-9.5	1.20	-3.5 (-8.4, 1.4)	0.39
n = 25/161 (16%)	HLP	12.1	3.9	15	-1.6	0.35			20.7	5.5	15	-6.0	0.76		
Hypertension	Exp	11.4	4.5	22	-1.9	0.42	-0.6 (-2.6, 1.4)	0.12	21.9	7.4	22	-8.4	1.06	-2.7 (-6.8, 1.4)	0.30
n = 40/161 (25%)	HLP	11.6	4.1	18	-1.3	0.29			23.4	7.3	18	-5.7	0.72		
IBS	Exp	11.9	4.7	33	-2.7	0.58	-0.2 (-2.0, 1.5)	0.04	24.7	7.3	33	-9.0	1.13	-3.2 (-6.6, 0.2)	0.36
n = 59/161 (37%)	HLP	13.7	4.0	26	-2.5	0.54			23.2	6.2	26	-5.8	0.73		
Migraine	Exp	10.4	6.1	12	-2.0	0.43	-1.2 (-3.8, 1.4)	0.25	22.9	10.2	12	-6.7	0.84	-0.6 (-6.1, 4.8)	0.07
n = 26/161 (16%)	HLP	13.1	4.8	14	-0.8	0.16			23.9	5.6	14	-6.1	0.76		
Osteoarthritis	Exp	11.5	3.9	24	-3.2	0.69	-1.5 (-3.7, 0.6)	0.31	22.9	7.3	24	-9.0	1.13	-4.1 (-8.5, 0.4)	0.46
n = 49/161 (30%)	HLP	10.8	4.4	25	-1.7	0.36			24.1	8.3	25	-4.9	0.62		
Osteoporosis	Exp	11.0	-	1	-1.1	0.24	-1.1 (-6.1, 3.9)	0.22	10.0	-	1	2.3	-0.29	7.8 (-1.7, 17.3)	-0.87
n = 5/161 (3%)	HLP	10.3	2.2	4	-0.03	0.01			30.0	8.1	4	-5.5	0.70		
Psoriasis	Exp	8.0	-	1	-1.8	0.40	-2.1 (-9.5, 5.3)	0.43	26.0	-	1	-4.7	0.59	-1.1 (-12.4, 10.2)	0.13
n = 6/161 (4%)	HLP	13.2	2.9	5	0.3	-0.07			24.6	8.8	5	-3.6	0.45		
SSD	Exp	10.6	4.9	37	-2.6	0.57	-0.2 (-1.9, 1.5)	0.05	25.7	7.9	37	-9.6	1.21	-1.2 (-4.6, 2.3)	0.13
n = 69/161 (43%)	HLP	12.7	4.5	32	-2.4	0.52			25.7	7.0	32	-8.4	1.06		

Thyroid disease (any)	Exp	12.6	6.1	16	-2.6	0.57	0.0 (-3.5, 3.6)	0.00	23.1	8.4	16	-9.0	1.14	-0.1 (-5.2, 5.0)	0.01
n = 24/161 (15%)	HLP	13.4	4.7	8	-2.7	0.58			20.5	6.9	8	-9.0	1.13		
Tinnitus	Exp	10.4	2.7	8	-5.0	1.09	-3.2 (-5.8, -0.6)	0.64	21.8	7.7	8	-11.3	1.43	-8.3 (-15.3, -1.3)	0.93
n = 13/161 (8%)	HLP	10.8	4.9	5	-1.8	0.39			21.4	11.7	5	-3.0	0.38		

Notes. Diagnoses self-reported by at least 5 participants, based on questions that probed a series of specific lifetime diagnoses given by a physician, or in the case of exhaustion disorder (approximately the same thing as clinical burnout, listed in the adapted Swedish version of the ICD-10) a physician or psychologist. Estimates derived from linear mixed effects regression models fitted on multiply imputed data. The scales were administered as self-report questionnaires via the Internet at the pre-treatment assessment (pre), each week over the 10-week treatment, at post-treatment assessment (post), and 6 and 12 months after the end of treatment (not reported here). Standardized within-group effects (*d*s) were calculated as the negated point estimate for the model-implied change (as listed under “est (95% CI)”), divided by the pre-treatment standard deviation for the entire sample in the non-imputed data (*N*=161). Standardized between-group effects (*d*s) were calculated as the point estimate for the model-implied difference (as listed under “est (95% CI)”), divided by the endpoint standard deviation for the entire sample in the non-imputed data. Exp, Internet-delivered exposure therapy; HLP, Internet-delivered healthy lifestyle promotion; IBS, irritable bowel syndrome; PHQ-15, Patient Health Questionnaire 15 rephrased to concern the past week only; SSD, Somatic symptom disorder according to the Diagnostic and Statistical Manual of Mental Disorders, 5th Edition; SSD-12, Somatic Symptom Disorder B-criteria scale 12 rephrased to concern the past week only; Tx, treatment condition.

Table DS6. Key dichotomous outcomes in Internet-delivered exposure therapy vs. standardized healthy lifestyle promotion for distress related to persistent physical symptoms

Outcome	Sample	Criterion	Treatment	Observed *n* (%)	OR (95% CI)
Beneficial outcomes					
Clinically significant improvement, reliability of 0.93	Pre PHQ-15 ≥ 10	ΔPHQ-15 < ca -3.4, post PHQ-15 < 10	Exp	24/41 (59%)	2.22 (1.12, 4.41)
			HLP	16/52 (31%)	
Clinically significant improvement, reliability of 0.65	Pre PHQ-15 ≥ 10	ΔPHQ-15 < ca -7.6, post PHQ-15 < 10	Exp	11/41 (27%)	1.65 (0.63, 4.35)
			HLP	7/52 (13%)	
Minimal clinically important improvement	Full sample	ΔPHQ-15 ≤ -3	Exp	47/74 (64%)	2.24 (1.16, 4.33)
			HLP	35/79 (44%)	
Unwanted outcomes					
Minimal clinically important deterioration	Full sample	ΔPHQ-15 ≥ 3	Exp	8/74 (11%)	1.46 (0.51, 4.14)
			HLP	7/79 (9%)	
At least one adverse event reported post-treatment	Full sample		Exp	16/74 (22%)	1.19 (0.55, 2.57)
			HLP	15/79 (19%)	

Notes. Odds ratios (ORs) and their confidence intervals were derived from logistic regression run on the multiply imputed data. Clinically significant improvement stands for whether the participant improved to an extent that, given the reliability of the outcome, can be assumed to indicative of true change on the level of the individual (with 95% confidence), and also scored below the clinical range (below 10) after treatment. Because there is uncertainty concerning the test-retest reliability of the PHQ-15, this analysis was repeated based on one relatively high test-retest reliability estimate and one relatively low test-retest reliability estimate identified in the existing literature, in accordance with the preregistered statistical analysis plan. Exp, Internet-delivered exposure therapy; HLP, Internet-delivered healthy lifestyle promotion; PHQ-15, Patient Health Questionnaire 15 rephrased to concern the past week only.

Table DS7. Mean scores in Internet-delivered exposure therapy vs. standardized healthy lifestyle promotion for distress related to persistent physical symptoms

**Outcome**	**Tx**	**Estimated means from mixed effects models**								**Means and *SD*s based on observed data**											
		**Pre**		**Post**		**6 m**		**12 m**		**Pre**			**Post**			**6 m**			**12 m**		
		M	***SE***	***M***	***SE***	***M***	***SE***	***M***	***SE***	***M***	***SD***	***n***	***M***	***SD***	***n***	***M***	***SD***	***n***	***M***	***SD***	***n***
Primary																					
Somatic symptom burden	Exp	10.6	0.5	7.9	0.5	8.3	0.5	9.0	0.6	11.1	4.7	80	7.4	4.8	74	8.0	4.4	73	8.6	4.8	68
PHQ-15	HLP	11.0	0.5	9.0	0.5	-	-	-	-	11.3	4.5	81	9.0	5.0	79	-	-	-	-	-	-
Secondary																					
Symptom preoccupation	Exp	22.6	0.9	13.2	1.1	14.5	1.2	15.3	1.1	24.0	8.3	80	12.4	8.9	74	13.9	9.9	73	14.4	8.8	68
SSD-12	HLP	22.3	0.9	15.6	1.1	-	-	-	-	23.3	7.6	81	15.0	8.7	79	-	-	-	-	-	-
Depression symptoms	Exp	7.7	0.6	5.3	0.5	5.2	0.5	6.1	0.6	7.7	4.8	80	5.1	4.0	74	5.0	3.9	73	5.9	5.1	68
PHQ-9	HLP	7.9	0.5	6.8	0.5	-	-	-	-	7.9	5.0	81	6.8	5.2	79	-	-	-	-	-	-
General anxiety	Exp	6.2	0.5	4.8	0.5	4.0	0.4	4.4	0.5	6.2	3.9	80	4.6	4.1	74	3.8	3.4	73	4.5	4.1	68
GAD-7	HLP	5.7	0.5	5.5	0.5	-	-	-	-	5.7	4.7	81	5.5	4.5	79	-	-	-	-	-	-
Functional impairment	Exp	24.2	1.8	17.1	1.9	15.6	2.0	17.2	2.1	24.2	16.7	80	15.5	14.9	74	15.0	17.3	73	15.9	15.9	68
WD2-12	HLP	26.7	1.8	20.8	1.9	-	-	-	-	26.7	16.2	81	21.0	17.1	79	-	-	-	-	-	-

Notes. Linear mixed effects regression models were fitted on multiply imputed data ($N = 161$). All scales were administered as self-report questionnaires via the Internet at the pre-treatment assessment (pre), post-treatment assessment (post), and 6 and 12 months after the end of treatment. For the 6-month follow-up analyses, a spline was added at the post-treatment assessment, to enable modelling of different rates of change for the main and follow-up phases. These models were run on the exposure data only as the healthy lifestyle promotion group was crossed over to exposure therapy after the post-treatment assessment. The PHQ-15 and SSD-12 were also administered each week over the 10-week treatment (between pre and post), thus resulting in 9 additional weekly assessments, which are not reported here. Exp, Internet-delivered exposure therapy; HLP, Internet-delivered healthy lifestyle promotion; PHQ-9, Patient Health Questionnaire 9; PHQ-15, Patient Health Questionnaire 15 rephrased to concern the past week only; SD, standard deviation; SE, standard error; SSD-12, Somatic Symptom Disorder B-criteria scale 12 rephrased to concern the past week only; Tx, treatment condition; WD2-12, 12-item World Health Organization Disability Assessment Schedule 2.0.

Table DS8. Moderator analyses pertaining to mean change in exposure therapy vs. standardized healthy lifestyle promotion

**Potential moderator variable**	**Scale /measure**	**Distribution**	**Hypothesis**[a]	**Moderation of treatment difference in pre-post change**	
		***M* (*SD*), median; range / *n* (%)**		**PHQ-15**	**SSD-12**
				**est (95% CI); *z***	**est (95% CI); *z***
Sociodemographics					
Age	Application data	48.2 (12.1), 51; 20-73	**no**	-0.02 (-0.1, 0.1); -0.56	-0.1 (-0.3, 0.1); -0.97
Female gender	Application data	137/161 (85%)	**no**	-1.3 (-4.1, 1.5); -0.88	-2.2 (-8.1, 3.7); -0.72
Post-secondary education	Application data	130/161 (81%)	exploratory	0.3 (-2.2, 2.8); 0.25	-0.9 (-6.1, 4.3); -0.33
Pre-treatment symptomatology					
Somatic symptom burden	PHQ-15	11.2 (4.6), 11; 0-27	**+**	**-0.3 (-0.5, -0.1); -2.42**[b]	**-0.7 (-1.2, -0.3); -3.06**
Sensitivity analysis: pain subscale	*PHQ-15 subscale*	*1.0 (0.5), 1; 0-2*	***as above***	***-2.3 (-4.3, -0.2); -2.16***	*-3.8 (-8.1, 0.5); -1.71*
Symptom preoccupation	SSD-12	23.6 (7.9), 24; 3-43	**+**	**-0.2 (-0.3, -0.1); -3.39**[b, c]	-0.1 (-0.4, 0.2); -0.75
Sensitivity analysis: health anxiety	*HAI-14*	*18.2 (5.9), 18; 4-38*	*as above*	*-0.1 (-0.3, 0.03); -1.54*	*0.1 (-0.2, 0.5); -0.62*
Depression core symptoms	PHQ-2	1.6 (1.5), 1; 0-6	-	-0.5 (-1.3, 0.2); -1.38	**-1.7 (-3.3, -0.1); -2.09**
Functional impairment	WD2-12	25.5 (16.4), 22.9; 0-68.8	+	-0.03 (-0.1, 0.03); -1.02	-0.1 (-0.2, 0.1); -0.88
Other clinical characteristics					
Years with somatic distress	Application data	11.9 (11.0), 9; 0-57	exploratory	-0.03 (-0.1, 0.1); -0.62	-0.03 (-0.2, 0.2); -0.30
Symptoms medically explained[d]	Application data	40/161 (25%)	**no**	0.4 (-1.9, 2.8); 0.37	0.8 (-4.1, 5.8); 0.34
Recruitment and treatment process					
Routine care (referred or listed)	Application data	42/161 (26%)	**no**	-1.7 (-4.0, 0.7); -1.41	-4.6 (-9.5, 0.3); -1.83
Completer (initiated ≥5 modules)[e]	Web platform	118/161 (73%)	+	0.5 (-1.9, 3.0); 0.40	1.3 (-4.1, 6.6); 0.47

Notes. All potential moderators were pre-specified and passed an initial test where these were required to have Pearson correlations less than 0.60. Estimates were derived from linear mixed effects regression models fitted on multiply imputed data, and moderation of the treatment difference in pre-post change was tested on the basis of the coefficient for the three-way interaction of time, condition, and potential moderator. All scales were administered as self-report questionnaires via the Internet at the pre-treatment assessment (pre), each week over the 10-week treatment, at post-treatment assessment (post), and 6 and 12 months after the end of treatment (not reported here). Exp, Internet-delivered exposure therapy; HLP, Internet-delivered healthy lifestyle promotion; PHQ-15, Patient Health Questionnaire 15 rephrased to concern the past week only; SSD-12, Somatic Symptom Disorder B-criteria scale 12 rephrased to concern the past week only.

[a] In the “Hypothesis” column, “+” indicates that a higher level on the potential moderator variable was expected to be associated with a larger effect of exposure therapy relative to healthy lifestyle promotion (a negative coefficient). Analogously, “-” indicates that a higher level on the potential moderator variable was expected to be associated with a smaller effect of exposure therapy relative to healthy lifestyle promotion (a positive coefficient). Bold lines indicate that the outcome corroborated the hypothesis.

[b] A model was run with both the PHQ-15 and SSD-12 as potential moderators. In this model, the moderation coefficient for the SSD-12 (est: -0.1; z=-2.02) but not the PHQ-15 (est: -0.2; z=-1.50) remained significant.

[c] A model was run with both the PHQ-15 and PHQ-2 as potential moderators. In this model, the moderation coefficient for the PHQ-15 (est: -0.6; *z*=-2.47) but not the PHQ-2 (est: -0.9; *z*=-1.05) remained significant.
[d] This variable was coded 0 for functional somatic syndromes and mixed cases.
[e] For this variable, moderation of within-group effects were also evaluated. On the PHQ-15, there was no significant moderation in exposure therapy (est: -0.1; *z*=-0.90) or healthy lifestyle promotion (est: -0.1; *z*=-1.32). On the SSD-12, there was no significant moderation in exposure therapy (est: -2.5; *z*=-1.40) or healthy lifestyle promotion (est: -3.8; *z*=-1.88).

Table DS9. Estimated treatment difference in mean change as a function of significant moderators

Moderator	Value	% of sample ≥ value	Estimated between-group effect			
			PHQ-15		SSD-12	
			est	*d*	est	*d*
PHQ-15	0	161/161 (100%)	-	-	5.3	-0.60
	5	151/161 (94%)	1.0	-0.20	1.7	-0.19
	10	99/161 (61%)	-0.4	0.08	-2.8	0.31
	15	37/161 (23%)	-1.8	0.37	-5.5	0.62
	20	4/161 (2%)	-3.2	0.65	-9.2	1.03
SSD-12	0	161/161 (100%)	4.5	-0.91	-	-
	15	143/161 (89%)	1.2	-0.25	-	-
	20	106/161 (66%)	0.1	-0.02	-	-
	25	69/161 (43%)	-1.0	0.20	-	-
	30	38/161 (24%)	-2.1	0.42	-	-
	35	18/161 (11%)	-3.2	0.64	-	-
	40	2/161 (1%)	-4.2	0.86	-	-
PHQ-2	0	161/161 (100%)	-	-	-0.2	0.02
	1	111/161 (70%)	-	-	-1.9	0.21
	2	80/161 (50%)	-	-	-3.6	0.41
	3	35/161 (22%)	-	-	-5.3	0.60
	4	20/161 (12%)	-	-	-7.1	0.79
	5	5/161 (3%)	-	-	-8.8	0.99

Notes. A negative mean difference (”est”), and positive standardized effect (”*d*”), is indicative of an average advantage of exposure vs. healthy lifestyle promotion. In this table, empty cells mirror the fact that the baseline PHQ-2 was not a significant moderator of the between-group effect on the PHQ-15, and the baseline SSD-12 was not a significant moderator of the between-group effect on the SSD-12 (see Table DS8). PHQ-15, Patient Health Questionnaire 15 rephrased to concern the past week only; SSD-12, Somatic Symptom Disorder B-criteria scale 12 rephrased to concern the past week only.

Table DS10. Estimated treatment difference in mean change in the Patient Health Questionnaire 15 as a function of moderators in a non-planned multivariable model

PHQ-15	SSD-12	Estimated between-group effect	
		PHQ-15	
		est	*d*
5	0	3.8	-0.76
5	15	1.6	-0.33
5	20	0.9	-1.19
5	25	0.2	-0.04
5	30	-0.5	0.10
5	35	-1.2	0.24
10	0	2.8	-0.57
10	15	0.7	-0.14
10	20	0.0	-0.00
10	25	-0.7	0.15
10	30	-1.4	0.29
10	35	-2.1	0.43
15	0	1.9	-0.38
15	15	-0.3	0.05
15	20	-1.0	0.19
15	25	-1.7	0.34
15	30	-2.4	0.48
15	35	-3.1	0.62
20	0	0.9	-0.19
20	15	-1.2	0.24
20	20	-1.9	0.38
20	25	-2.6	0.53
20	30	-3.3	0.67
20	35	-4.0	0.81

Notes. A negative mean difference (”est”), and positive standardized effect (”*d*”), is indicative of an average advantage of exposure vs. healthy lifestyle promotion. PHQ-15, Patient Health Questionnaire 15 rephrased to concern the past week only; SSD-12, Somatic Symptom Disorder B-criteria scale 12 rephrased to concern the past week only.

Table DS11. Estimated treatment difference in mean change in the Somatic Symptom Disorder B-criteria scale 12 as a function of moderators in a non-planned multivariable model

PHQ-15	PHQ-2	Estimated between-group effect	
		SSD-12	
		est	*d*
0	0	5.7	-1.16
0	1	4.8	-0.97
0	2	3.8	-0.78
0	3	2.9	-0.59
0	4	2.0	-0.40
0	5	1.0	-0.21
5	0	2.6	-0.52
5	1	1.6	-0.33
5	2	0.7	-1.14
5	3	-0.2	0.05
5	4	-1.2	0.24
5	5	-2.1	0.43
10	0	-0.6	0.11
10	1	-1.5	0.30
10	2	-2.4	0.49
10	3	-3.4	0.68
10	4	-4.3	0.87
10	5	-5.3	1.06
15	0	-3.7	0.75
15	1	-4.6	0.94
15	2	-5.6	1.13
15	3	-6.5	1.32
15	4	-7.5	1.51
15	5	-8.4	1.70
20	0	-6.9	1.39
20	1	-7.8	1.58
20	2	-8.7	1.76
20	3	-9.7	1.95
20	4	-10.6	2.14
20	5	-11.5	2.33

Notes. A negative mean difference (”est”), and positive standardized effect (”*d*”), is indicative of an average advantage of exposure vs. healthy lifestyle promotion. PHQ-15, Patient Health Questionnaire 15 rephrased to concern the past week only; SSD-12, Somatic Symptom Disorder B-criteria scale 12 rephrased to concern the past week only.

Table DS12 Adverse events as reported in the post-treatment assessment.

**Categories of adverse events**	**Treatment**	**Observed *n* (%)**
Increased symptom severity	Exp	16/74 (22%)
	HLP	13/79 (16%)
Dissatisfaction with the quality of treatment	Exp	2/74 (3%)
	HLP	0/79 (0%)
Experience of dependency on the treatment or therapist	Exp	0/74 (0%)
	HLP	0/79 (0%)
Stigma as a consequence of participation	Exp	0/74 (0%)
	HLP	0/79 (0%)
Feelings of hopelessness	Exp	0/74 (0%)
	HLP	3/79 (4%)
Lowered self-esteem or a sense of failure	Exp	2/74 (3%)
	HLP	3/79 (4%)

Notes. In the post-treatment assessment, if applicable, patients were asked to report up to three adverse events that they perceived could have been related to their participation in the trial, and to elaborate on any such experiences in free text. Adverse events were categorized by a person blind to treatment condition according to categories of commonly reported adverse events derived from factor analysis (Rozental et al. 2016). The number and proportions represent patients reporting at least one adverse event per category, among the subsample which completed the post-treatment assessment. Exp, Internet-delivered exposure therapy; HLP, Internet-delivered healthy lifestyle promotion.

Table DS13 Planned test of potential gender effect on exclusions due to primary psychiatric disorder

**Reason for exclusion**	**Men**	**Women**
	***n* (%)**	***n* (%)**
Applicant excluded due to severe or clearly primary psychiatric condition	16 (30%)	63 (40%)
Applicant excluded for another reason	37 (70%)	94 (60%)

Notes. In line with the preregistered analysis plan, we tested the hypothesis that men are more inclined than women to seek health care for somatic symptoms when they are in fact suffering from a psychiatric condition. This test was based on a 2x2 $\chi 2$ test of exclusions due to another principal psychiatric condition vs. exclusions due to other factors, men vs. women. Applicants who withdrew their application or could not be reached were not included in this test. The test was not statistically significant ($\chi 2 = 1.67$, $p = 0.197$).

References

Axelsson E, Andersson E, Ljótsson B, Wallhed Finn D and Hedman E (2016) The Health Preoccupation Diagnostic Interview: Inter-rater reliability of a structured interview for diagnostic assessment of DSM-5 somatic symptom disorder and illness anxiety disorder. *Cognitive Behaviour Therapy* **45**(4)**,** 259-269. https://doi.org/10.1080/16506073.2016.1161663.

Bailer J, Kerstner T, Witthöft M, Diener C, Mier D and Rist F (2016) Health anxiety and hypochondriasis in the light of DSM-5. *Anxiety Stress Coping* **29**(2)**,** 219-239. https://doi.org/10.1080/10615806.2015.1036243.

Berman AH, Bergman H, Palmstierna T and Schlyter F (2005) Evaluation of the Drug Use Disorders Identification Test (DUDIT) in criminal justice and detoxification settings and in a Swedish population sample. *European Addiction Research* **11**(1)**,** 22-31. https://doi.org/10.1159/000081413.

Burton C, Fink P, Henningsen P, Löwe B, Rief W and Euronet-Soma Group (2020) Functional somatic disorders: discussion paper for a new common classification for research and clinical use. *BMC Medicine* **18,** 34. https://doi.org/10.1186/s12916-020-1505-4.

Claassen-van Dessel N, van der Wouden JC, Twisk JWR, Dekker J and van der Horst HE (2018) Predicting the course of persistent physical symptoms: Development and internal validation of prediction models for symptom severity and functional status during 2years of follow-up. *Journal of Psychosomatic Research* **108,** 1-13. https://doi.org/10.1016/j.jpsychores.2018.02.009.

Han C, Pae CU, Patkar AA, Masand PS, Kim KW, Joe SH and Jung IK (2009) Psychometric properties of the Patient Health Questionnaire-15 (PHQ-15) for measuring the somatic symptoms of psychiatric outpatients. *Psychosomatics* **50**(6)**,** 580-585. https://doi.org/10.1176/appi.psy.50.6.580.

Hybelius J, Gustavsson A, af Winklerfelt Hammarberg S, Toth-Pal E, Johansson R, Ljótsson B and Axelsson E (2022) A unified Internet-delivered exposure treatment for undifferentiated somatic symptom disorder: single-group prospective feasibility trial. *Pilot Feasibility Stud* **8**(1)**,** 149. https://doi.org/10.1186/s40814-022-01105-0.

Hybelius J, Kosic A, Salomonsson S, Wachtler C, Wallert J, Nordin S and Axelsson E (2024) Measurement Properties of the Patient Health Questionnaire-15 and Somatic Symptom Scale-8: A Systematic Review and Meta-Analysis. *JAMA Netw Open* **7**(11)**,** e2446603. https://doi.org/10.1001/jamanetworkopen.2024.46603.

Häuser W, Bialas P, Welsch K and Wolfe F (2015) Construct validity and clinical utility of current research criteria of DSM-5 somatic symptom disorder diagnosis in patients with fibromyalgia syndrome. *Journal of Psychosomatic Research* **78**(6)**,** 546-552. https://doi.org/10.1016/j.jpsychores.2015.03.151.

Jacobson NS and Truax P (1991) Clinical significance: a statistical approach to defining meaningful change in psychotherapy research. *Journal of Consulting and Clinical Psychology* **59**(1)**,** 12-19. https://doi.org/10.1037/0022-006x.59.1.12.

Kohlmann S, Löwe B and Shedden-Mora MC (2018) Health Care for Persistent Somatic Symptoms Across Europe: A Qualitative Evaluation of the EURONET-SOMA Expert Discussion. *Front Psychiatry* **9,** 646. https://doi.org/10.3389/fpsyt.2018.00646.

Kroenke K, Spitzer RL and Williams JB (2002) The PHQ-15: validity of a new measure for evaluating the severity of somatic symptoms. *Psychosomatic Medicine* **64**(2)**,** 258-266. https://doi.org/10.1097/00006842-200203000-00008.

Körber S, Frieser D, Steinbrecher N and Hiller W (2011) Classification characteristics of the Patient Health Questionnaire-15 for screening somatoform disorders in a primary care setting. *Journal of Psychosomatic Research* **71**(3)**,** 142-147. https://doi.org/10.1016/j.jpsychores.2011.01.006.

Liao SC, Huang WL, Ma HM, Lee MT, Chen TT, Chen IM and Gau SS (2016) The relation between the patient health questionnaire-15 and DSM somatic diagnoses. *BMC Psychiatry* **16**(1)**,** 351. https://doi.org/10.1186/s12888-016-1068-2.

Ludvigsson JF, Otterblad-Olausson P, Pettersson BU and Ekbom A (2009) The Swedish personal identity number: possibilities and pitfalls in healthcare and medical research. *European Journal of Epidemiology* **24**(11)**,** 659-667. https://doi.org/10.1007/s10654-009-9350-y.

Rozental A, Kottorp A, Boettcher J, Andersson G and Carlbring P (2016) Negative Effects of Psychological Treatments: An Exploratory Factor Analysis of the Negative Effects Questionnaire for Monitoring and Reporting Adverse and Unwanted Events. *PloS One* **11**(6)**,** e0157503. https://doi.org/10.1371/journal.pone.0157503.

Saunders JB, Aasland OG, Babor TF, de la Fuente JR and Grant M (1993) Development of the Alcohol Use Disorders Identification Test (AUDIT): WHO Collaborative Project on Early Detection of Persons with Harmful Alcohol Consumption - II. *Addiction* **88**(6)**,** 791-804. https://doi.org/10.1111/j.1360-0443.1993.tb02093.x.

Sheehan DV, Lecrubier Y, Sheehan KH, Amorim P, Janavs J, Weiller E, Hergueta T, Baker R and Dunbar GC (1998) The Mini-International Neuropsychiatric Interview (M.I.N.I.): the development and validation of a structured diagnostic psychiatric interview for DSM-IV and ICD-10. *Journal of Clinical Psychiatry* **59 Suppl 20,** 22-33.

Smith RC and Dwamena FC (2007) Classification and diagnosis of patients with medically unexplained symptoms. *Journal of General Internal Medicine* **22**(5)**,** 685-691. https://doi.org/10.1007/s11606-006-0067-2.

van Buuren S and Groothuis-Oudshoorn K (2011) mice: Multivariate imputation by chained equations in R. *Journal of Statistical Software* **45**.
